# Notch1-promoted TRPA1 expression in erythroleukemic cells suppresses erythroid but enhances megakaryocyte differentiation

**DOI:** 10.1038/srep42883

**Published:** 2017-02-21

**Authors:** Ji-Lin Chen, Yueh-Hsin Ping, Min-Jen Tseng, Yuan-I Chang, Hsin-Chen Lee, Rong-Hong Hsieh, Tien-Shun Yeh

**Affiliations:** 1 Department and Institute of Pharmacology, School of Medicine, National Yang-Ming University, Taipei 112, Taiwan; 2 Institute of Anatomy and Cell Biology, School of Medicine, National Yang-Ming University, Taipei 112, Taiwan; 3 Department of Life Science, National Chung Cheng University, Chia-Yi 621, Taiwan; 4 Department and Institute of Physiology, School of Medicine, National Yang-Ming University, Taipei 112, Taiwan; 5 School of Nutrition and Health Sciences, College of Nutrition, Taipei Medical University, Taipei 110, Taiwan; 6 Institute of Biochemistry and Molecular Biology, National Yang-Ming University, Taipei, Taiwan; 7 Genome Research Center, National Yang-Ming University, Taipei 112, Taiwan; 8 Graduate Institute of Medical Sciences, College of Medicine, Taipei Medical University, Taipei 110, Taiwan

## Abstract

The Notch1 pathway plays important roles in modulating erythroid and megakaryocyte differentiation. To screen the Notch1-related genes that regulate differentiation fate of K562 and HEL cells, the expression of transient receptor potential ankyrin 1 (TRPA1) was induced by Notch1 receptor intracellular domain (N1IC), the activated form of Notch1 receptor. N1IC and v-ets erythroblastosis virus E26 oncogene homolog 1 (Ets-1) bound to TRPA1 promoter region to regulate transcription in K562 cells. Transactivation of TRPA1 promoter by N1IC depended on the methylation status of TRPA1 promoter. N1IC and Ets-1 suppressed the DNA methyltransferase 3B (DNMT3B) level in K562 cells. Inhibition of TRPA1 expression after Notch1 knockdown could be attenuated by nanaomycin A, an inhibitor of DNMT3B, in K562 and HEL cells. Functionally, hemin-induced erythroid differentiation could be suppressed by TRPA1, and the reduction of erythroid differentiation of both cells by N1IC and Ets-1 occurred *via* TRPA1. However, PMA-induced megakaryocyte differentiation could be enhanced by TRPA1, and the surface markers of megakaryocytes could be elevated by nanaomycin A. Megakaryocyte differentiation could be reduced by Notch1 or Ets-1 knockdown and relieved by TRPA1 overexpression. The results suggest that Notch1 and TRPA1 might be critical modulators that control the fate of erythroid and megakaryocyte differentiation.

The Notch pathway regulates several biological functions, including proliferation, differentiation, apoptosis, and tumorigenesis^1^,^2^; exerts complex and multi-faceted functions; and plays either oncogenic or tumor-suppressive roles in tumorigenesis^3^,^4^. The Notch pathway also acts as a critical regulator of multiple developmental processes, including hematopoiesis^1^,^5^,^6^. Mounting lines of evidence have suggested that activation of the Notch1 pathway modulates erythroid^7^,^8^,^9^ and megakaryocyte^8^ differentiation. In mammals, evolutionarily conserved Notch signaling is composed of four Notch receptor paralogues (Notch1–4) and five Notch ligands of two families^1^,^3^,^4^. Notch receptors are activated upon ligand binding and are subsequently to release their intracellular domains, the activated forms of Notch receptors. The intracellular domain subsequently translocates into the nucleus to modulate target gene expression *via* mechanisms both dependent and independent of C promoter binding factor-1 (CBF1)/recombination signal binding protein-Jκ (RBP-Jκ)^1^,^3^,^4^.

The transient receptor potential (TRP) ankyrin 1 (TRPA1), also known as ANKTM1, is a calcium-permeable non-selective ion channel of the TRP superfamily^10^,^11^ and is a transformation-sensitive protein originally cloned from human lung fibroblasts^12^. Previous reports have demonstrated that TRPA1 expression was restricted to sensory neurons^10^. However, TRPA1 was also detected in several tissues, including but not limited to the brain, heart, lung, skeletal muscle, small intestine, colon, and pancreas of humans^13^. In sensory neurons, TRPA1 co-localizes with substance P, transient receptor potential vanilloid 1 (TRPV1), and calcitonin gene-related peptide^14^,^15^. A range of environmental pungents or irritants such as mustard oil (allyl isothiocyanate, AITC), cinnamon oil, acrolein, allicin, methylparaben, and formalin can activate TRPA1^16^. Intracellular Ca^2+^ directly activates TRPA1 through a putative EF-hand calcium binding domain at the N-terminal of TRPA1^17^,^18^. Additionally, TRPA1 also responds to a variety of endogenous agonists associated with inflammation and oxidative stress. For example, inflammatory mediators bradykinin and prostaglandins can indirectly activate TRPA1 *via* second messengers and kinases^19^,^20^,^21^. The oxidant agents produced by inflammation and oxidative stress, which include 4-hydroxynonenal, hydrogen peroxide, and hypochloride, are able to activate TRPA1^22^,^23^. Expression of TRPA1 is closely linked to levels of pro-inflammatory cytokines. Deletion of glycoprotein 130 (the subunit of interleukin-6 receptor) down-regulates TRPA1 expression in small sensory neurons^24^. Tumor necrosis factor-α and interleukin-1α induce TRPA1 levels in human fibroblast-like synoviocytes^25^. Erythropoiesis can be repressed by pro-inflammatory cytokines such as tumor necrosis factor-α, leading to anemia in several diseases, including chronic inflammatory disease, myelodysplastic syndrome, and cancer^26^.

In the current study, we identified TRPA1 as one of the Notch1 pathway-induced genes in K562 and HEL erythroleukemia cells. To date, no report exists on the role and molecular mechanism of TRPA1 in controlling the development of myeloid lineage. Thus, the involvement of Notch1 pathway-mediated TRPA1 expression in erythroid and megakaryocyte differentiation was investigated in this work.

## Results

### N1IC induced TRPA1 expression in a CBF1-independent manner

**Figure 1:**
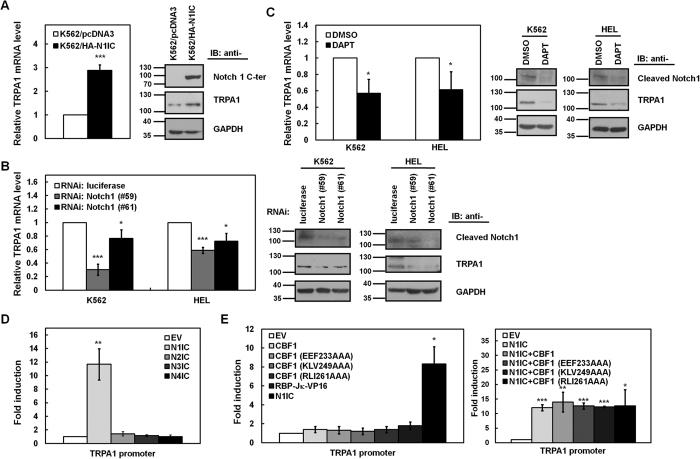
N1IC induced TRPA1 expression in a CBF1-independent manner. (**A**) The relative transcription levels of TRPA1 in N1IC-expressing K562/HA-N1IC and control (K562/pcDNA3) cells were determined by quantitative real-time PCR and normalized to those of β-actin (*left*). Whole-cell extracts of K562/HA-N1IC and control cells were prepared and analyzed by Western blot analysis using anti-Notch1 C-terminal (C-ter), anti-TRPA1, and anti-GAPDH antibodies (*right*). (**B**) After transfection of siRNA vectors against Notch1 receptor (#59 and #61) or luciferase into K562 and HEL cells for two days, transcription levels of TRPA1 in the transfected cells were measured by quantitative real-time PCR (*left*). Whole-cell extracts of transfected cells were also prepared for Western blot analysis using anti-cleaved Notch1, anti-TRPA1, and anti-GAPDH antibodies (*right*). (**C**) K562 and HEL cells were treated with 50 μM DAPT or an equal volume of vehicle (DMSO) for 24 hours. Transcription levels of TRPA1 in the treated cells were measured by quantitative real-time PCR (*left*). Whole-cell extracts of the treated cells were also prepared for Western blot analysis using anti-cleaved Notch1, anti-TRPA1, and anti-GAPDH antibodies (*right*). The full-length blots are presented in Supplementary Fig. S12. (**D**) Reporter plasmid −1,057/−12 containing TRPA1 promoter (nucleotides −1,057 to −12) was co-transfected with expression constructs of Notch1 receptor (N1IC), Notch2 receptor (N2IC), Notch3 receptor (N3IC), and Notch4 receptor (N4IC) intracellular domains or empty vector (EV) into K562 cells for 48 hours for reporter gene assay. (**E**) Reporter plasmid −1,057/−12 was co-transfected with expression constructs of wild-type CBF1, CBF1 mutants (EEF233AAA, KLV249AAA, and RLI261AAA), constitutively active RBP-Jκ-VP16 fusion protein (RBP-JκVP16), and N1IC or empty vector into K562 cells for reporter gene assay (*left*). Reporter plasmid −1,057/−12 was co-transfected with expression construct of N1IC and expression plasmids wild-type CBF1 or CBF1 mutants into K562 cells for reporter gene assay (*right*). The means of three independent experiments performed in triplicate are shown. *P < 0.05; **P < 0.01; ***P < 0.001.

To screen the Notch1 pathway-related genes that control the development of myeloid lineage, quantitative real-time PCR analyses were performed using previously established K562 cells expressing Notch1 receptor intracellular domain (N1IC) with an NH_2_-terminal hemagglutinin (HA) tag (K562/HA-N1IC) and their control cells (K562/pcDNA3), as previously described^27^. TRPA1, one of the differentially expressed genes, showed elevated transcript (Fig. 1A, *left*) and protein (Fig. 1A, *right*) levels in K562/HA-N1IC cells compared with K562/pcDNA3 control cells. To confirm the effect of endogenous Notch1 receptor on TRPA1 expression, K562 and HEL erythroleukemia cells were transfected with siRNA vectors against Notch1 receptor (#59 and #61) for blockage of the Notch1 pathway. Furthermore, cells were also treated with N-[N-(3,5-difluorophenacetyl)-L-alanyl]-S-phenylglycine *t*-butyl ester (DAPT), an inhibitor of γ-secretase which is essential for activation of Notch pathways. Quantitative real-time PCR and Western blot analyses showed that both mRNA and protein levels of TRPA1 in K562 and HEL cells were reduced after Notch1 receptor knockdown (Fig. 1B and Supplementary Fig. S1) or DAPT treatment (Fig. 1C).

To determine whether all four Notch pathways affect TRPA1 expression at the transcriptional level, expression constructs of the intracellular domains of four Notch receptors were co-transfected with a reporter plasmid containing the TRPA1 promoter into K562 cells for reporter gene assays. The data showed that only exogenous N1IC significantly enhanced TRPA1 promoter activity in K562 cells (Fig. 1D and Supplementary Fig. S2). To clarify whether N1IC-mediated transactivation of TRPA1 promoter activity depends on CBF1, the reporter gene assay was used in K562 cells after co-transfecting the reporter plasmid containing TRPA1 promoter and constructs of wild-type CBF1 or three CBF1 mutants, including RLI261AAA expressing normal nuclear staining and EEF233AAA and KLV249AAA expressing cytosolic staining^28^. The ectopic expression of wild-type CBF1 or CBF1 mutants did not significantly affect TRPA1 promoter activity compared with N1IC transfection (Fig. 1E). Furthermore, transfection with the expression construct of the constitutively active RBP-Jκ-VP16 fusion protein did not notably change the TRPA1 promoter activity (Fig. 1E). These results suggested that TRPA1 promoter activity is induced by the Notch1 pathway primarily in a CBF1-independent manner.

### N1IC enhanced TRPA1 promoter activity partially through v-ets erythroblastosis virus E26 oncogene homolog 1 (Ets-1)

**Figure 2:**
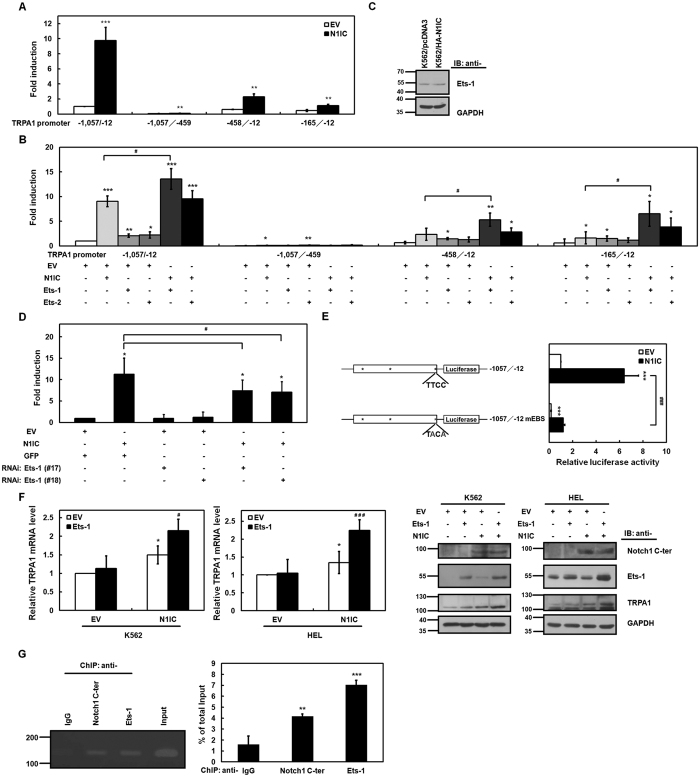
N1IC enhanced TRPA1 promoter activity partially through Ets-1. (**A**) Reporter plasmids (−1,057/−12, −1,057/−459, −458/−12, and −165/−12) containing various lengths of DNA fragments of TRPA1 promoter were co-transfected with N1IC-expressing construct (N1IC) or empty vector (EV) into K562 cells for reporter gene assay. (**B**) Reporter plasmids (−1,057/−12, −1,057/−459, −458/−12, and −165/−12) were co-transfected with expression constructs of N1IC, Ets-1, and Ets-2 or empty vector into K562 cells for reporter gene assay. (**C**) Whole-cell extracts of the N1IC-expressing K562/HA-N1IC and control (K562/pcDNA3) cells were prepared for Western blot analysis using anti-Ets-1 and anti-GAPDH antibodies. (**D**) Reporter plasmid −1,057/−12 was co-transfected with N1IC-expressing construct (N1IC) or empty vector and siRNA vectors against Ets-1 (#17 and #18) or its pPGK-GFP control vector (GFP) into K562 cells for reporter gene assay. (**E**) Schematic representation of luciferase reporter plasmids (−1,057/−12 and −1,057/−12 mEBS) containing TRPA1 promoter (*left*). Stars indicate positions of putative Ets-1-binding sites. The core sequence (nucleotides −40 to −37) of the putative Ets-1-binding site in the −1,057/−12 mEBS reporter plasmid was mutated from TTCC to TACA. Reporter plasmids −1,057/−12 and −1,057/−12 mEBS were co-transfected with N1IC-expressing construct (N1IC) or empty vector into K562 cells for reporter gene assay (*right*). (**F**) N1IC-expressing construct (N1IC) was co-transfected with Ets-1-expressing construct (Ets-1) or empty vector into K562 and HEL cells for two days. Transcription levels of TRPA1 in the transfected cells were measured by quantitative real-time PCR (*left*). Whole-cell extracts of the transfected cells were also prepared for Western blot analysis using anti-Notch1 C-terminal (C-ter), anti-Ets-1, anti-TRPA1, and anti-GAPDH antibodies (*right*). The full-length blots are presented in Supplementary Fig. S12. (**G**) N1IC-expressing K562/HA-N1IC cells were harvested for ChIP assay using anti-IgG, anti-Notch1 C-terminal (C-ter), and anti-Ets-1 antibodies. The immunoprecipitated DNAs were used to amplify the 134-bp PCR products of TRPA1 promoter (*left*). Percentages of the immunoprecipitated DNAs were quantified by quantitative real-time PCR and normalized to total input DNA (*right*). The means of at least three independent experiments performed in triplicate are shown. The full-length gel is presented in Supplementary Fig. S12. *P < 0.05; **P < 0.01; ***P < 0.001. ^#^*P* < 0.05; ^###^*P* < 0.001.

To map the critical transactivated regions of the TRPA1 promoter by N1IC, reporter plasmids containing DNA fragments of different regions in the human TRPA1 promoter were constructed for a luciferase reporter gene assay (Fig. 2A). Reporter gene activities in K562 cells were measured after co-transfecting N1IC-expressing construct and reporter plasmids containing different regions of the TRPA1 promoter. The plasmids containing the region of nucleotides −1,057 to −12 showed approximately 10-fold induction, and regions of nucleotides −458 to −12, and −165 to −12 presented lower induction activities. However, no induction was observed from the plasmid containing the region of nucleotides −1,057 to −459. Therefore, the DNA fragment from nucleotides −165 to −12 of the TRPA1 promoter is sufficient for its transactivation by N1IC.

Using the search engines PROMO^29^ and JASPAR^30^ to analyze transcription factor-binding sites in human TRPA1 promoter, three putative binding sites were found for Ets-1. Ets-1 is involved in regulating the development of myeloid lineage in hematopoietic progenitor cells^31^ and specification as well as development of T cells^32^. To investigate whether Ets-1 participates in N1IC-transactivated TRPA1 promoter activity in K562 cells, ectopic Ets-1 was either overexpressed by transfecting the Ets-1 expression construct (Supplementary Fig. S3A) or endogenous Ets-1 was knocked down by transfecting siRNA vectors against Ets-1 (#17 and #18) (Supplementary Fig. S3B). Luciferase reporter gene assays were performed in K562 cells after co-transfection with reporter plasmids containing DNA fragments of different regions in the TRPA1 promoter and expression constructs of N1IC, Ets-1, and Ets-2 (which is closely related to Ets-1 in amino acid sequence) (Fig. 2B). N1IC-transactivated TRPA1 promoter activity was further enhanced by overexpression of Ets-1 but not by Ets-2. N1IC overexpression in K562/HA-N1IC cells did not alter the Ets-1 level compared with K562/pcDNA3 control cells (Fig. 2C). The reporter gene assay also showed that Ets-1 knockdown could partially suppress N1IC-transactivated TRPA1 promoter activity in K562 cells (Fig. 2D).

One putative Ets-1 binding site exists in the region of nucleotides −165 to −12 of the human TRPA1 promoter. To clarify whether N1IC-transactivated TRPA1 promoter activity depends on this Ets-1-binding site, the core sequence located at nucleotides −40 to −37 of TRPA1 promoter was mutated from TTCC to TACA. After transfection of the reporter plasmid containing TRPA1 promoter into K562 cells, the TRPA1 promoter activity was significantly decreased with the construct of the mutated Ets-1-binding site (Fig. 2E). The N1IC-enhanced activity of the TRPA1 promoter was also diminished after co-transfection of the N1IC-expressing construct and a reporter plasmid containing the TRPA1 promoter with a mutated Ets-1-binding site into K562 cells. The data demonstrated that the Ets-1-binding site located at nucleotides −40 to −37 of the TRPA1 promoter is essential for N1IC-mediated transactivation of promoter activity. Moreover, quantitative real-time PCR and Western blot analyses showed that the N1IC-enhanced mRNA and protein levels of TRPA1 in K562 and HEL cells were further promoted by co-transfecting the Ets-1-expressing construct (Fig. 2F).

We surmised that N1IC and Ets-1 cooperatively bind to the DNA of the TRPA1 promoter to up-regulate transcription in the context of living cells. The DNA-binding abilities of N1IC and Ets-1 on the TRPA1 promoter in N1IC-expressing K562/HA-N1IC cells were examined *via* the chromatin immunoprecipitation (ChIP) assay using anti-Notch1 C-terminal and anti-Ets-1 antibodies (Fig. 2G). The results of the ChIP assay showed that N1IC and Ets-1 bound to the TRPA1 promoter in the chromosomal DNAs of K562/HA-N1IC cells.

### N1IC-transactivated TRPA1 promoter activity depended on methylation of the TRPA1 promoter

**Figure 3:**
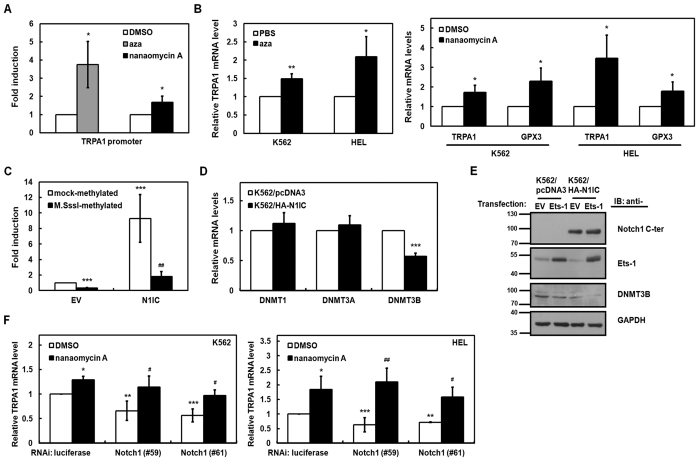
N1IC-transactivated TRPA1 promoter activity depended on methylation of TRPA1 promoter. (**A**) After transfection with reporter plasmid −1,057/−12 containing TRPA1 promoter for 24 hours, K562 cells were treated with 5 μM 5-azacytidine (aza), 30 nM nanaomycin A, or vehicle (DMSO) for 24 hours for reporter gene assay. (**B**) The transcription levels of TRPA1 or glutathione peroxidase 3 (GPX3) (a gene down-regulated by promoter hypermethylation in cancers) in K562 and HEL cells treated with 5-azacytidine (*left*) or nanaomycin A (*right*) were measured by quantitative real-time PCR. (**C**) N1IC-expressing construct (N1IC) or empty vector (EV) were co-transfected with M.SssI-methylated or mock-methylated reporter plasmid −1,057/−12 containing TRPA1 promoter into K562 cells for reporter gene assay. (**D**) The transcription levels of DNA methyltransferases DNMT1, DNMT3A, and DNMT3B in N1IC-expressing K562/HA-N1IC and control cells (K562/pcDNA3) were determined by quantitative real-time PCR. (**E**) After transfection with Ets-1-expressing construct (Ets-1) or empty vector into K562/HA-N1IC and K562/pcDNA3 cells, whole-cell extracts of the transfected cells were prepared for Western blot analysis using anti-Notch1 C-terminal (C-ter), anti-Ets-1, anti-DNMT3B, and anti-GAPDH antibodies. The full-length blots are presented in Supplementary Fig. S12. (**F**) After transfection with siRNA vectors against Notch1 receptor (#59 and #61) or luciferase and treatment with nanaomycin A or DMSO treatment for 48 hours, the transcription levels of TRPA1 in K562 (*left*) and HEL (*right*) cells were measured by quantitative real-time PCR. The means of three independent experiments performed in triplicate are shown. *P < 0.05; **P < 0.01; ***P < 0.001. ^#^*P* < 0.05; ^##^*P* < 0.01.

It has been reported that the methylation level of the TRPA1 promoter in the whole-blood DNA methylation pattern is associated with pain sensitivity^33^. After transfecting the reporter plasmid containing the TRPA1 promoter into K562 cells, the reporter gene activity was enhanced by treatment with 5-azacytidine, a DNA methyltransferases (DNMTs) inhibitor (Fig. 3A). Levels of TRPA1 mRNAs in K562 and HEL cells were up-regulated by 5-azacytidine treatment according to quantitative real-time PCR analyses (Fig. 3B, *left*). When the reporter plasmid containing the TRPA1 promoter was methylated with M.SssI, the reporter gene activity was reduced (Fig. 3C). The N1IC-enhanced activity of TRPA1 promoter was significantly reduced after co-transfecting the N1IC-expressing construct and M.SssI-treated reporter plasmid containing the TRPA1 promoter. These results suggest that N1IC-mediated transactivation of the TRPA1 promoter activity possibly depends on the methylation status of the TRPA1 promoter.

To delineate whether N1IC regulates the expression of DNMTs, which in turn modulate methylation of the TRPA1 promoter, quantitative real-time PCR analyses in K562 cells were performed (Fig. 3D). The results showed that N1IC overexpression decreased the mRNA levels of DNMT3B but not DNMT1 or DNMT3A in N1IC-expressing K562/HA-N1IC cells compared with K562/pcDNA3 control cells. The DNMT3B protein level in K562/HA-N1IC cells was also reduced by N1IC overexpression (Fig. 3E), and this reduced level was further suppressed by Ets-1 overexpression. Treatment with nanaomycin A, a selective DNMT3B inhibitor, increased the activity of a luciferase reporter gene containing the TRPA1 promoter in K562 cells (Fig. 3A) and also increased levels of TRPA1 mRNAs in K562 and HEL cells (Fig. 3B, *right*). To study whether N1IC induces TRPA1 expression through DNMT3B methyltransferase, quantitative real-time PCR analyses were used with K562 (Fig. 3F, *left*) and HEL (Fig. 3F, *right*) cells transfected with siRNA vectors against Notch1 (#59 and #61) and subsequently treated with nanaomycin A. The TRPA1 mRNA levels suppressed by Notch1 knockdown were restored by nanaomycin A treatment.

### TRPA1 suppressed the erythroid differentiation abilities of K562 and HEL cells

**Figure 4:**
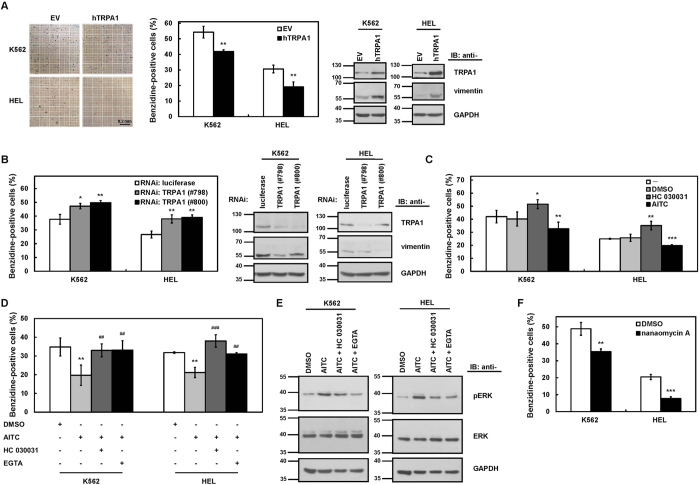
TRPA1 suppressed erythroid differentiation abilities of K562 and HEL cells. (**A**) K562 and HEL cells were transfected with TRPA1-expressing construct (hTRPA1) or empty vector (EV) for 48 hours. The transfected cells were treated with hemin for induction of erythroid differentiation, and the benzidine-positive cells were counted under light microscopy after staining (*left* and *middle*). Scale bar: 0.2 mm. Whole-cell extracts of the transfected cells were also prepared for Western blot analysis using anti-TRPA1, anti-vimentin, and anti-GAPDH antibodies (*right*). (**B**) K562 and HEL cells were transfected with siRNA vectors against TRPA1 (#798 and #800) or luciferase for 48 hours. As described above, the transfected cells were treated with hemin and used to count the percentages of benzidine-positive cells (*left*). Whole-cell extracts of the transfected cells were also prepared for Western blot analysis using anti-TRPA1, anti-vimentin, and anti-GAPDH antibodies (*middle* and *right*). (**C**) K562 and HEL cells were treated with an equal volume of 10 μM HC 030031, 5 μM AITC, DMSO, or PBS (−) and processed for erythroid differentiation by 40 μM hemin for 2 and 4 days, respectively. The benzidine-positive cells were counted as described above. (**D**) In the presence of 10 μM HC 030031 or 2 mM EGTA for 30 min, K562 and HEL cells were subsequently treated with 5 μM AITC or DMSO in combination with hemin to induce erythroid differentiation. (**E**) In the presence or absence of HC 030031 or EGTA for 30 min as described above, K562 (*left*) and HEL (*right*) cells were treated with AITC or DMSO for 5 min. Whole-cell extracts of the treated cells were prepared for Western blot analysis using anti-pERK, anti-ERK, and anti-GAPDH antibodies. The full-length blots are presented in Supplementary Fig. S12. (**F**) K562 and HEL cells were treated with nanaomycin A or DMSO in combination with hemin for 2 and 4 days, respectively. As described above, the benzidine-positive cells were counted. Values represent the means from three independent experiments. *P < 0.05; **P < 0.01; ***P < 0.001. ^##^*P* < 0.01; ^###^*P* < 0.001.

Previous reports demonstrated that N1IC^7^,^8^ and Ets-1^31^,^34^,^35^,^36^,^37^,^38^,^39^,^40^ are involved in the regulation of erythroid and megakaryocyte differentiation. To investigate whether the transactivation of TRPA1 by N1IC and Ets-1 as demonstrated in Fig. 2 also participates in erythroid differentiation, K562 and HEL cells were transfected with a TRPA1-expressing construct. Erythroid differentiation could be induced by hemin and quantified using benzidine-stained cells. The erythroid differentiation abilities of K562 and HEL cells, as represented by benzidine-stained positive cells, were significantly suppressed by TRPA1 overexpression (Fig. 4A, *left*). Additionally, overexpression of TRPA1 decreased CD235a-positive cells in hemin-treated K562 cells which were stained with antibodies and further analyzed by flow cytometry (Supplementary Fig. S4). The data showed that erythroid differentiation of K562 and HEL cells was suppressed by TRPA1 overexpression. It has been found that vimentin is down-regulated during erythroid differentiation^41^,^42^. When TRPA1 is overexpressed, Western blot analyses showed that vimentin was up-regulated in K562 and HEL cells (Fig. 4A, *right*). In contrast, the erythroid differentiation abilities were enhanced by TRPA1 knockdown after transfecting siRNA vectors against TRPA1 (#798 and #800) into K562 and HEL cells (Fig. 4B, *left*) and the vimentin levels were down-regulated by TRPA1 knockdown in both cells (Fig. 4B, *right*).

K562 and HEL cells were also treated with HC 030031, a selective TRPA1 antagonist, or AITC, a TRPA1 agonist, and hemin was subsequently added to induce erythroid differentiation. In line with the above observations, the erythroid differentiation abilities of the treated cells were increased by HC 030031 treatment but decreased by AITC treatment (Fig. 4C). TRPA1 activation led to ERK phosphorylation through calcium influx in small cell lung cancer cells^43^. However, the function of ERK activation in erythroid differentiation was previously demonstrated using different models with conflicting results^44^,^45^,^46^,^47^. To examine whether the pharmacological activation of TRPA1 by AITC inhibits erythroid differentiation through calcium influx, K562 and HEL cells were pretreated with HC 030031 for blockage of AITC-evoked calcium influx or with ethylene glycol-bis(2-aminoethylether)-N,N,N′,N′-tetraacetic acid (EGTA) for chelation of extracellular calcium prior to AITC treatment (Fig. 4D). Pretreatment with HC 030031 or EGTA reversed the effect of AITC-mediated suppression of the erythroid differentiation abilities of K562 and HEL cells. Moreover, the AITC-enhanced levels of ERK phosphorylation in K562 and HEL cells were also inhibited by pretreatment with HC 030031 or EGTA (Fig. 4E). In addition, treatment with the DNMT3B inhibitor nanaomycin A to increase TRPA1 expression also decreased the erythroid differentiation abilities of K562 and HEL cells (Fig. 4F).

### N1IC and Ets-1 reduced the erythroid differentiation abilities of K562 and HEL cells via TRPA1

**Figure 5:**
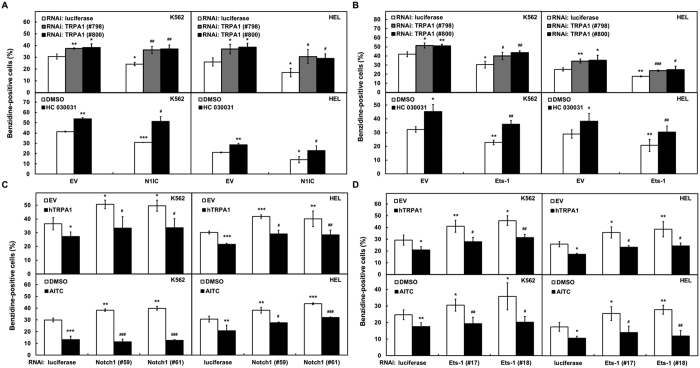
N1IC and Ets-1 reduced erythroid differentiation abilities of K562 and HEL cells *via* TRPA1. (**A,B**) K562 and HEL cells were co-transfected with expression constructs of N1IC (**A**) and Ets-1 (**B**) or empty vector (EV) and siRNA vectors against TRPA1 (#798 and #800) or luciferase for 2 days. Hemin-induced erythroid differentiation of the transfected cells was performed as described in Fig. 4 (*upper*). The cells transfected with expression constructs of N1IC (**A**) and Ets-1 (**B**) or empty vector were also treated with HC 030031 or DMSO in combination with hemin to induce erythroid differentiation (*lower*). (**C,D**) K562 and HEL cells were co-transfected with siRNA vectors against Notch1 receptor (**C**) and Ets-1 (**D**) or luciferase and TRPA1-expressing construct (hTRPA1) or empty vector for 2 days. As described above, erythroid differentiation of the transfected cells was performed after hemin treatment (*upper*). The cells transfected with siRNA vectors against Notch1 receptor (**C**) and Ets-1 (**D**) or luciferase were also treated with AITC or DMSO in combination with hemin to induce erythroid differentiation (*lower*). Values represent the means from at least three independent experiments. *P < 0.05; **P < 0.01; ***P < 0.001. ^#^*P* < 0.05; ^##^*P* < 0.01; ^###^*P* < 0.001.

To further examine whether N1IC and Ets-1 modulate erythroid differentiation through TRPA1, hemin-induced erythroid differentiation was applied in N1IC or Ets-1 overexpressed K562 and HEL cells in combination with TRPA1 knockdown or HC 030031 treatment. The reduction of erythroid differentiation abilities by overexpression of N1IC (Fig. 5A) or Ets-1 (Fig. 5B) could be restored after co-transfecting siRNA vectors against TRPA1 (#798 and #800) or HC 030031 treatment. Conversely, the enhancement of erythroid differentiation abilities by knocking down Notch1 (Fig. 5C) or Ets-1 (Fig. 5D) could be suppressed after co-transfecting with TRPA1-expressing construct or treating with AITC in K562 or HEL cells.

### TRPA1 promoted the megakaryocyte differentiation abilties of K562 and HEL cells

**Figure 6:**
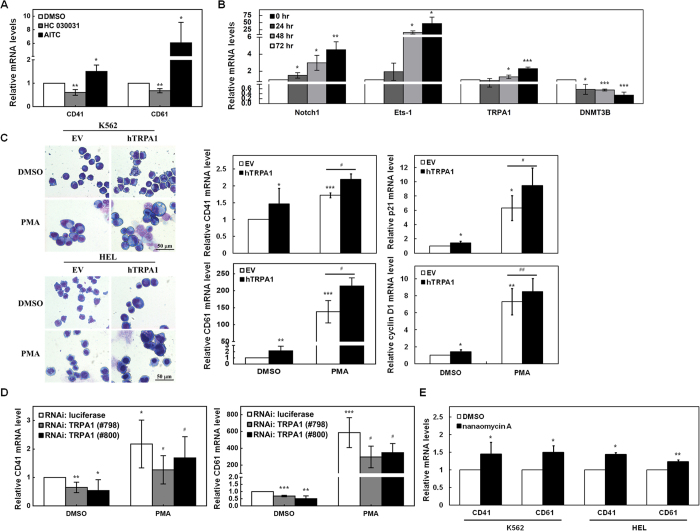
TRPA1 promoted megakaryocyte differentiation abilities of K562 cells. (**A**) After treatment with 10 μM HC 030031, 5 μM AITC, or DMSO for 2 days, the transcription levels of CD41 and CD61 in K562 cells were determined by quantitative real-time PCR. (**B**) Quantitative real-time PCR was used to detect the transcription levels of Notch1 receptor, Ets-1, TRPA1, and DNMT3B in K562 cells treated with 5 ng/ml PMA at the time indicated. (**C**) K562 and HEL cells transfected with TRPA1-expressing construct (hTRPA1) or empty vector (EV) were treated with PMA or DMSO for 2 days to induce megakaryocytic differentiation. The morphological changes in the treated cells were examined under light microscopy after Giemsa staining (*left*). Scale bar: 50 μm. The transcription levels of CD41, CD61, p21, and cyclin D1 of the treated cells were also measured by quantitative real-time PCR (*right*). The original images are presented in Supplementary Fig. S13. (**D**) K562 cells were treated with PMA or DMSO for induction of megakaryocyte differentiation as described above after transfection with siRNA vectors against TRPA1 (#798 and #800) or luciferase. Quantitative real-time PCR was performed to detect the transcription levels of CD41 (*left*) and CD61 (*right*). (**E**) After treating K562 and HEL cells with nanaomycin A or DMSO for 2 days, the transcript levels of CD41 and CD61 were measured by quantitative real-time PCR. The means of at least three independent experiments performed in triplicate are shown. *P < 0.05; **P < 0.01; ***P < 0.001. ^#^*P* < 0.05; ^##^*P* < 0.01.

In addition to erythroid differentiation, the effect of TRPA1 on megakaryocyte differentiation was also surveyed. The mRNA levels of CD41 and CD61 (the differentiation surface markers of megakaryocytes) in K562 cells were suppressed by HC 030031 treatment but enhanced by AITC treatment (Fig. 6A). To explore the roles of TRPA1 in megakaryocyte differentiation, K562 and HEL cells were treated with phorbol 12-myristate 13-acetate (PMA) to induce megakaryocyte differentiation. After PMA treatment in K562 cells, quantitative real-time PCR showed that the mRNA expression of Notch 1 receptor, Ets-1, and TRPA1 was up-regulated, but in contrast, that of DNMT3B was down-regulated in a time-dependent manner (Fig. 6B). Distinct morphological changes were observed upon microscopic examination of K562 and HEL cells after PMA treatment and Giemsa staining (Fig. 6C, *left*). The PMA-treated cells showed multiple phenotypes of megakaryocyte differentiation, including an increase in cell size, polyploidization, and the presence of vacuoles. Morphologically, TRPA1 promoted the PMA-induced megakaryocyte differentiation abilities of K562 and HEL cells. Overexpression of TRPA1 slightly caused the arrest of cell cycle and increase of PMA-mediated polyploidization in K562 cells (Supplementary Fig. S5). Additionally, significantly elevated expression of CD41 and CD61 mRNAs were noted in K562 cells treated with PMA, according to quantitative real-time PCR analyses (Fig. 6C, *middle*). This increase was further elevated after transfection with a TRPA1-expressing construct. Overexpression of TRPA1 also increased CD41-positive cells in PMA-treated K562 cells which were stained with antibodies and further analyzed by flow cytometry (Supplementary Fig. S6). The PMA-promoted mRNA levels of p21^48^ and cyclin D1^49^, which induce polyploidization of megakaryocytes, were further increased after transfection with the TRPA1-expressing construct (Fig. 6C, *right*). Therefore, the typical characters of megakaryocyte differentiation became more definite when these cells were transfected with the TRPA1-expressing construct. Alternatively, K562 and HEL cells were transfected with siRNA vectors against TRPA1 (#798 and #800) following PMA treatment for megakaryocyte differentiation. The treated cells were examined by microscope after Giemsa staining (Supplementary Fig. S7) and harvested for quantitative real-time PCR analyses (Fig. 6D). The elevation of CD41 (Fig. 6D, *left*) and CD61 (Fig. 6D, *right*) mRNA levels in PMA-treated K562 cells was diminished by TRPA1 knockdown. Furthermore, the mRNA levels of CD41 and CD61 in K562 and HEL cells were increased by nanaomycin A treatment, which increased TRPA1 expression (Fig. 6E).

### N1IC and Ets-1 induced the megakaryocyte differentiation abilities of K562 and HEL cells through TRPA1

**Figure 7:**
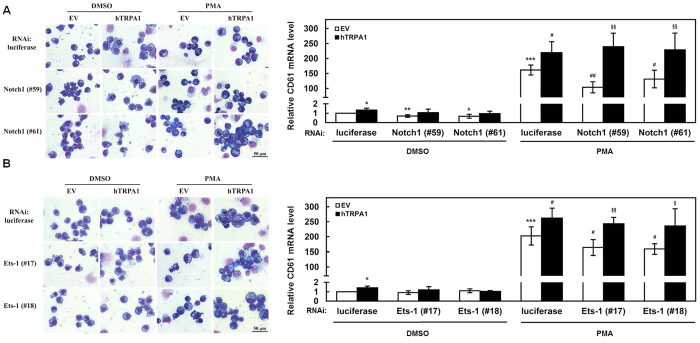
Reduction of megakaryocyte differentiation abilities in K562 and HEL cells by Notch1 or Ets-1 knockdown was relieved by TRPA1 overexpression. (**A**) After co-transfection with TRPA1-expressing construct (hTRPA1) or empty vector (EV) and siRNA vectors against Notch1 receptor (#59 and #61) or luciferase, K562 cells were treated with PMA or DMSO for induction of megakaryocyte differentiation and were subsequently subjected to morphological examination under light microscopy after Giemsa staining as described in Fig. 6 (*left*). The transcription levels of CD61 in the transfected cells were detected by quantitative real-time PCR (*right*). (**B**) K562 cells were co-transfected with TRPA1-expressing construct or empty vector and siRNA vectors against Ets-1 (#17 and #18) or luciferase. The transfected cells were treated with PMA or DMSO for induction of megakaryocyte differentiation as described above. Subsequently, the treated cells were used in morphological examination as described above (*left*) and for detection of transcript levels of CD61 by quantitative real-time PCR (*right*). The original images are presented in Supplementary Fig. S13. Scale bar: 50 μm. Values represent the means from at least three independent experiments. *P < 0.05; **P < 0.01; ***P < 0.001. ^#^*P* < 0.05; ^##^*P* < 0.01. ^§^*P* < 0.05; ^§§^*P* < 0.01.

To further decipher whether N1IC and Ets-1 also regulate megakaryocyte differentiation through TRPA1, K562 and HEL cells were treated with PMA after co-transfection with TRPA1-expressing construct and siRNA vectors against Notch1 receptor (#59 and #61) or Ets-1 (#17 and #18). Morphologically, knockdown of endogenous Notch1 receptor or Ets-1 inhibited the PMA-induced megakaryocyte differentiation abilities of K562 (Fig. 7A, *left*; Fig. 7B, *left*) and HEL (Supplementary Fig. S8) cells. The megakaryocyte differentiation abilities suppressed by knocking down Notch1 receptor and Ets-1 could be attenuated by overexpressing TRPA1. Moreover, quantitative real-time PCR analyses showed that the increase in CD61 mRNA expression in PMA-treated K562 cells was suppressed by Notch1 receptor (Fig. 7A, *right*) or Ets-1 (Fig. 7B, *right*) knockdown. However, this suppression of CD61 mRNA expression in megakaryocyte differentiation could be relieved by overexpression of TRPA1. In addition, K562 and HEL cells were treated with PMA for megakaryocyte differentiation after co-transfecting siRNA vectors against TRPA1 (#798 and #800) and expressing constructs of N1IC (Supplementary Fig. S9) or Ets-1 (Supplementary Fig. S10). The forced expression of Notch1 receptor and Ets-1 enhanced PMA-induced megakaryocyte differentiation of K562 and HEL cells, as shown by morphological examination. This enhancement of megakaryocyte differentiation abilities by Notch1 receptor and Ets-1 overexpression could be inhibited by TRPA1 knockdown.

### DNMT3B knockdown decreased abilities of erythroid differentiation but increased abilities of megakaryocyte differentiation in K562 cells

**Figure 8:**
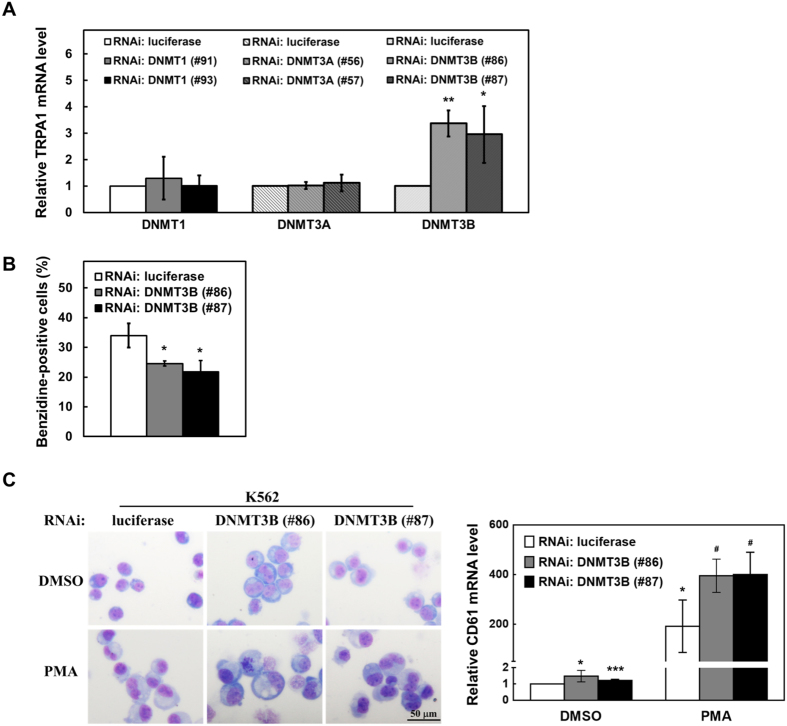
DNMT3B knockdown decreased abilities of erythroid differentiation but increased abilities of megakaryocyte differentiation in K562 cells. K562 cells were transfected with siRNA vectors against DNMT1 (#91 and #93), DNMT3A (#56 and #57), DNMT3B (#86 and #87) or luciferase for two days. (**A**) The relative transcription levels of TRPA1 in the transfected cells were determined by quantitative real-time PCR and normalized to those of β-actin. (**B**) The transfected cells were treated with 40 μM hemin for two days to induce erythroid differentiation, and the benzidine-positive cells were counted under light microscopy after staining. (**C**) The transfected cells were also treated with 5 ng/ml PMA for two days to induce megakaryocyte differentiation. The morphological changes in the treated cells were examined under light microscopy after Giemsa staining (*left*). The original images are presented in Supplementary Fig. S13. The transcription levels of CD61 of the treated cells were also measured by quantitative real-time PCR (*right*). The means of at least three independent experiments performed in triplicate are shown. *p < 0.05; **P < 0.01; ***P < 0.001. ^#^*P* < 0.05.

DNMT3B methyltransferase is involved in control of the N1IC-transactivated TRPA1 expression as demonstrated in Fig. 3F. To investigate whether DNMTs also regulates abilities of erythroid and megakaryocyte differentiation, K562 cells were transfected with siRNA vectors against DNMT1 (#91 and #93), DNMT3A (#56 and #57), or DNMT3B (#86 and #87) (Supplementary Fig. S11). Quantitative real-time PCR analyses showed that mRNA levels of TRPA1 were increased by DNMT3B knockdown but not by DNMT1 or DNMT3A knockdown (Fig. 8A). Furthermore, DNMT3B knockdown decreased abilities of hemin-induced erythroid differentiation in the transfected K562 cells (Fig. 8B). The transfected K562 cells were also treated with PMA and examined by microscope after Giemsa staining. Morphologically, DNMT3B knockdown increased the PMA-induced megakaryocyte differentiation abilities of K562 (Fig. 8C, *left*). Quantitative real-time PCR analyses showed that the increase of CD61 mRNA expression in the PMA-treated K562 cells was further elevated by DNMT3B knockdown (Fig. 8C, *right*).

## Discussion

To delineate the mechanism underlying Notch1 pathway-mediated differentiation of erythroid and megakaryocyte, TRPA1 was identified herein as a Notch1 pathway-induced gene in erythroleukemia cells. TRPA1 is widely expressed outside the central nervous system in humans^13^. The expression of TRPA1 is modulated by pro-inflammatory cytokines^24^,^25^, which regulate normal and diseased hematopoiesis^26^,^50^,^51^. This study scrutinized the roles of Notch1 pathway-enhanced TRPA1 in the development of myeloid lineage and showed that TRPA1 suppressed erythroid differentiation but enhanced megakaryocyte differentiation. To the best of our knowledge, this is the first report on the linkage between the Notch1 pathway and TRPA1 in controlling hematopoiesis.

The mechanism involved in regulating TRPA1 transcription is unclear thus far. Pro-inflammatory cytokines tumor necrosis factor-α and interleukin-1α induced TRPA1 levels in human fibroblast-like synoviocytes through NF-κB and HIF1α^25^. Based on the data in Figs 1 and 2, N1IC-enhanced TRPA1 promoter activity depended on Ets-1 but not on CBF1. Enrichment of overlapping Ets-1-binding sites near Notch1 receptor-binding sites was identified using ChIP deep sequencing in the genomes of human and murine T-lymphoblastic leukemia cells^52^. Furthermore, binding of N1IC to the CBF1-binding site and Ets-1 to Ets-1-binding site cooperatively activated rat renin promoter^53^. Therefore, expression of the N1IC-regulated gene is likely to be influenced by Ets-1 binding to an Ets-1-binding site near an N1IC-binding site.

The TRPA1 promoter is hypermethylated, and its DNA methylation level is correlated with pain sensitivity^33^. Until now, the relationship between methylation of the TRPA1 promoter and the Notch1 pathway or Ets-1 has remained poorly understood. It was documented that the Notch1 pathway slightly lessened the level of *de novo* methyltransferase DNMT3B and impaired the association of maintenance methyltransferase DNMT1 with the promoter of the cortical glial fibrillary acidic protein in neural precursor cells^54^. Ets-1 bound to the demethylated region in the forkhead-box protein P3 (Foxp3) locus and contributed to stabilization of Foxp3 expression in regulatory T cells^55^. The results of this study showed that the parallel stimulatory effect of N1IC and Ets-1 on transactivation of TRPA1 promoter activity depended on down-regulation of the DNMT3B level in K562 cells (Fig. 3).

The interrelationship of N1IC, Ets-1, and TRPA1 in regulating the development of myeloid lineage was analyzed. Based on the obtained data, N1IC and Ets-1 reduced erythroid differentiation (Fig. 5) but induced megakaryocyte differentiation (Fig. 7) of K562 and HEL cells partially through TRPA1. Previous studies found that N1IC suppressed hemin-induced erythroid differentiation of K562 cells^7^,^8^. However, previous results on the roles of N1IC in TPA-induced megakaryocyte differentiation of K562 cells were varied. Lam *et al*.^7^ reported that N1IC did not inhibit TPA-induced megakaryocyte differentiation in K562 cells. Ishiko *et al*.^8^ demonstrated that N1IC inhibited TPA-induced megakaryocyte differentiation in K562 cells, whereas Roy *et al*.^56^ showed that N1IC induced early megakaryopoiesis in K562 or HEL cells. In different systems of hematopoietic stem cells, several reports suggested that Notch signaling activated by different Notch ligands enhanced megakaryocyte differentiation^57^,^58^,^59^,^60^, whereas others indicated the opposite effect^8^,^61^. Additionally, Ets-1 was also involved in the development of myeloid lineage in several studies with contradictory results. It is debatable whether Ets-1 promoted^35^,^38^ or inhibited^31^,^34^,^36^,^37^ erythroid differentiation. In hematopoietic progenitor cells, Ets-1 was up-regulated during megakaryocyte differentiation^39^, and its overexpression augmented megakaryocyte differentiation^31^. It was also speculated that TRPA1 function was not relevant for PMA-induced maturation of human MEG-01 megakaryoblastic cells^62^. However, the maturation of MEG-01 cells in this study was evaluated only by levels of Rap1b protein, a ubiquitous Ras-related GTPase, which was increased after megakaryocytic maturation.

Hematopoiesis is a complicated process regulated by a signaling cascade in a stage-specific manner. For example, ERK activation could be linked to erythroid^47^,^63^ and megakaryocyte^64^,^65^ differentiation in K562 cells. It has been demonstrated that TRPA1 activation led to calcium- and Src-dependent stimulation of ERK1/2 in human small cell lung cancer cells^43^. Proliferator-activated receptor gamma ligand 15d-PGJ_2_, an endogenous TRPA1 agonist^66^, promoted megakaryoblastic maturation and platelet release of MEG-01 cells^67^. Functions of megakaryocytes are regulated by both calcium mobilization from intracellular stores and extracellular calcium entry^68^. Notably, TRPA1 regulates store-operated Ca^2+^ entry (SOCE), the primary Ca^2+^ influx pathway in non-excitable mammalian cells, by modulating STIM1-Orai1 association in MEG-01 megakaryoblastic cells^62^. Our data in the current study showed that TRPA1 up-regulated p21, cyclin D1, CD41, and CD61 levels, and cell polyploidization in K562 and HEL cells after PMA treatment (Fig. 6).

Leukemic transformation is related to dysregulation of proliferation, differentiation, or cell death in hematopoietic cells. Differentiation therapy is a potentially less toxic form of cancer therapy that uses drugs, alone or in combination, to modify differentiation and growth in cancer cells^69^. Evidence suggested that differentiation therapy might offer promise for improved cancer therapy^69^. It has also been shown that treatment with differentiation-inducing small molecules overcomes self-renewal in neoplastic pluripotent stem cell models of human cancer^70^. Thus, treatment with differentiation inducers might be capable of inducing the differentiation of cancer stem cells and entering normal cellular life cycles to inhibit survival of cancer stem cells and tumor generation. Moreover, anemia is a complication that affects prognosis and quality of life in patients with cancers and chronic inflammatory diseases^26^. Thrombocytopenia might be caused by chemotherapy and radiation treatment in patients with cancer and can result in significant morbidity and mortality^67^. Therefore, studies on the mechanisms involved in control of erythroid and megakaryocyte differentiation are essential for creating therapeutic benefits in the treatment of anemia and thrombocytopenia, respectively. Based on our data (Figs 4, 5, 6, 7), the Notch1 pathway, Ets-1, and TRPA1 were involved in control of erythroid and megakaryocyte differentiation in K562 and HEL cells. The strategies for Notch pathway modulation were tested in preclinical evaluation and clinical trials^4^. TRPA1 antagonists were also developed^71^,^72^ and subsequently underwent clinical trials^73^. In the current study, the cross talk among the Notch1 pathway, Ets-1, and TRPA1 in erythroid and megakaryocyte differentiation suggests a potential combinatorial strategy for treatment of hematological malignancies in the near future.

## Materials and Methods

### Plasmids and plasmid construction

The plasmid hTRPA1 contains cDNA of human TRPA1^22^. Expression constructs pcDNA-HA-N1IC, pcDNA-myc-N2IC-His, pcDNA-N3IC-myc-His, and pcDNA-N4IC-myc-His express the intracellular domains of human Notch1–4 receptors, respectively^74^. Expression plasmids pcDNA-CBF1-myc-His^75^, pSG5Flag-RBPVP16^76^, RLI261AAA, EEF233AAA, and KLV249AAA^28^ contain cDNAs of wild-type or mutant CBF1s. Constructs pCI-Ets-1 and pCI-Ets-2 contain the cDNAs encoding Ets-1 and Ets-2, respectively^77^.

The DNA fragments of human TRPA1 promoter were amplified by PCR from the genomic DNA of K562 cells and then constructed in front of the luciferase gene in pGL3-Basic vector. Reporter plasmids −1,057/−12, −1,057/−459, −458/−12, and −165/−12 contain various lengths of TRPA1 promoter from nucleotide −1,057 to −12, −1,057 to −459, −458 to −12, and −165 to −12, respectively. Reporter plasmid −1,057/−12 mEBS contains the TRPA1 promoter DNA fragment from nucleotide −1,057 to −12 in which the putative Ets-1-binding site located at nucleotide −40 to −37 was mutated from TTCC to TACA. Reporter plasmid 4 × wtCBF1Luc contains four copies of CBF1-response elements^78^. All constructs were verified by DNA sequencing.

To knock down the endogenous TRPA1, Notch1, Ets-1, and DNMTs, the target sequences listed in the Supplementary Tables S1 were constructed in the small interfering RNA (siRNA) vector pLKO.1 as described^79^. The siRNA vector against luciferase (pLKO.1-shLuc) was used as a negative control for knockdown validation. The expression construct pPGK-GFP contains cDNA encoding the green fluorescent protein (GFP) in the pLKO.1 vector was used as a control for luciferase reporter gene assay.

### *In vitro* DNA methylation

Following the manufacturer’s protocol, 1 μg of reporter plasmid −1,057/−12 containing TRPA1 promoter was incubated with 1 unit of CpG Methyltransferase (M.SssI, New England BioLabs), supplemented with 160 μM S-adenosylmethionine at 37 °C for 4 hours. Reaction was terminated by heating at 65 °C for 20 minutes, and then the methylated plasmid DNA was purified.

### Cell culture and plasmid transfection

Human erythroleukemia K562 and HEL cells were cultured in RPMI 1640 medium with 10% fetal bovine serum. The stable K562 cells expressing the HA-N1IC fusion protein (K562/HA-N1IC) and their control cells (K562/pcDNA3) were described previously^27^. K562 and HEL cells were transiently transfected with plasmids by transfection reagent PolyJet^TM^ (SignaGen Laboratories). For luciferase reporter gene assay, cells were seeded onto 6-well plates at 5 × 10^5^ cells/well and subsequently transfected for 2 days. Luciferase activity was measured using the Dual-Luciferase^TM^ Reporter Assay System (Promega) and then normalized with *Renilla* luciferase activity for transfection efficiency.

All HC 030031 (TOCRIS), nanaomycin A (BioVision), AITC, DAPT, 5-azacytidine, and PMA (Sigma-Aldrich) at the indicated concentration in dimethyl sulfoxide (DMSO) or an equal volume of DMSO were added for treatment.

### Western blot analysis

Whole-cell extracts were prepared and then resolved by sodium dodecyl sulfate-polyacrylamide gel electrophoresis as described^27^. Western blot analyses were performed with anti-Notch1 C terminal, anti-Ets-1, anti-DNMT3B (Santa Cruz), anti-cleaved Notch1, anti-ERK1/2, anti-phospho-ERK1/2 (Cell Signaling Technology), anti-TRPA1 (Novus Biologicals), anti-vimentin (Thermo Fisher Scientific), and anti-GAPDH (Genetex) antibodies.

### ChIP assay

To cross-link DNA and protein, K562/HA-N1IC cells were treated with formaldehyde (1% final concentration) at room temperature for 15 minutes as described previously^27^. Reaction was terminated by adding glycine to a final concentration of 0.125 M. To amplify the DNA fragment in chromosomal DNAs, nuclear extracts were prepared for the succeeding ChIP assay using protein A Sepharose-conjugated antibodies of anti-IgG, anti-Notch1 C terminal, and anti-Ets-1 antibodies at 4 °C for 16 hours. Then the immunoprecipitated DNAs were used to amplify the 134-bp PCR products of DNA fragment of TRPA1 promoter by specific primers (Supplementary Tables S1).

### Quantitative real-time PCR analysis

According to the manufacturer’s protocol, total RNA was extracted using the Trizol reagent (Invitrogen) and then cDNA was synthesized by Moloney murine leukemia virus reverse transcriptase (New England BioLabs) with an oligo (dT)18 primer. For mRNA detection, the cDNAs were amplified with primers (Supplementary Tables S1) using an ABI StepOne Plus system with SYBR Green Master Mix (Applied Biosystems) as described^9^. The relative quantification of mRNA levels was normalized with those of β-actin.

### Erythroid differentiation and benzidine staining

The transfected K562 and HEL cells were treated with 40 μM hemin (Sigma-Aldrich) to induce erythroid differentiation for 2 days and 4 days, respectively, as described^9^. Then the cells (1 × 10^5^) were washed with ice-cold phosphate-buffered saline and subsequently resuspended in ice-cold phosphate-buffered saline. The 0.2% (wt/vol) benzidine dihydrochloride (Sigma-Aldrich) was prepared in 0.5 M glacial acetic acid. The cells were incubated at room temperature for 10 min after addition of benzidine dihydrochloride solution (0.1% [wt/vol] final concentration) containing hydrogen peroxide (0.3% final concentration). The benzidine-positive cells with dark-blue staining were quantified under light microscopy.

### Megakaryocyte differentiation and Giemsa staining

To induce megakaryocyte differentiation, K562 and HEL cells were treated with 5 ng/ml PMA (Sigma-Aldrich) for 2 days. The cells (5 × 10^4^) were fixed onto a microscope slide with methanol for 5 minutes, and then air-dried. Then the slide was stained with Giemsa stain for 15 minutes as described by the supplier (Sigma-Aldrich) and cell morphology was examined under light microscopy.

### Statistical analyses

Statistical calculations were performed using an independent Student’s t-test for simple comparison of two groups. The difference was considered statistical significance if the P value was less than 0.05.

## Additional Information

**How to cite this article:** Chen, J.-L. *et al*. Notch1-promoted TRPA1 expression in erythroleukemic cells suppresses erythroid but enhances megakaryocyte differentiation. *Sci. Rep.* **7**, 42883; doi: 10.1038/srep42883 (2017).

**Publisher's note:** Springer Nature remains neutral with regard to jurisdictional claims in published maps and institutional affiliations.

## Supplementary Material

Supplementary Information
