# Supplementary material for: Notch1-promoted TRPA1 expression in erythroleukemic cells suppresses erythroid but enhances megakaryocyte differentiation
Source: Sci Rep. 2017 Feb 21;7:42883. doi: 10.1038/srep42883 (PMC5318885; doi:10.1038/srep42883)
Supplement: Supplementary Information [file srep42883-s1.pdf]

Title:

Notch1-promoted TRPA1 expression in erythroleukemic cells suppresses erythroid but enhances megakaryocyte differentiation

Ji-Lin Chen[1,2], Yueh-Hsin Ping[1], Min-Jen Tseng[3], Yuan-I Chang[4], Hsin-Chen Lee[1], Rong-Hong Hsieh[5], Tien-Shun Yeh[2,6,7,8]

[1]Department and Institute of Pharmacology, School of Medicine, National Yang-Ming University, Taipei 112, Taiwan, [2]Institute of Anatomy and Cell Biology, School of Medicine, National Yang-Ming University, Taipei 112, Taiwan, [3]Department of Life Science, National Chung Cheng University, Chia-Yi 621, Taiwan, [4]Department and Institute of Physiology, School of Medicine, National Yang-Ming University, Taipei 112, Taiwan, [5]School of Nutrition and Health Sciences, College of Nutrition, Taipei Medical University, Taipei 110, Taiwan, [6]Institute of Biochemistry and Molecular Biology, National Yang-Ming University, Taipei, Taiwan, [7]Genome Research Center, National Yang-Ming University, Taipei 112, Taiwan, [8]Graduate Institute of Medical Sciences, College of Medicine, Taipei Medical University, Taipei 110, Taiwan

Supplementary Table, Figures, and Figure legends

Table S1. Sequence of primers for siRNA, PCR, and real-time PCR

Assays		Sequence (5′ to 3′)	Amplicon (bp)
siRNA	Notch1 (#59)	CTTTGTTTCAGGTTCAGTATT	
	Notch1 (#61)	GCCGAACCAATACAACCCTCT	
	Ets-1 (#17)	ATCCCGCTATACCTCGGATTA	
	Ets-1 (#18)	GACCGTGCTGACCTCAATAAG	
	TRPA1 (#798)	CCAGGCAATAAATGTCCAATT	
	TRPA1 (#800)	CCTCCGAAACTTCAACATGAT	
	DNMT1 (#91)	GCCCAATGAGACTGACATCAA	
	DNMT1 (#93)	CGACTACATCAAAGGCAGCAA	
	DNMT3A (#56)	GCCTCAGAGCTATTACCCAAT	
	DNMT3A (#57)	CCAGATGTTCTTCGCTAATAA	
	DNMT3B (#86)	CCATGCAACGATCTCTCAAAT	
	DNMT3B (#87)	GCAGGCAGTAGGAAATTAGAA	
real-time PCR	TRPA1	F GAGAGTCCTTCCTAGAACCATATCTGA	104
		R CATGAGGACAATTGGGACAAATATT	
	Notch1	F CACTGTGGGCGGGTCC	85
		R GTTGTATTGGTTCGGCACCAT	
	Ets-1	F TCACTAAAGAACAGCAACGA	92
		R ATTCACAGCCCACATCAC	
	DNMT1	F TACCTGGACGACCCTGACCTC	101
		R TTGGCATCAAAGATGGACAGC	
	DNMT3A	F TGATGAGCGCACAAGAGAGC	105
		R TGTTCCAGGGTAACATTGAGGC	
	DNMT3B	F GGCAAGTTCTCCGAGGTCTCT	113
		R TGGTACATGGCTTTTCGATAGGA	
	CD41	F GGTGAGAGGGAGCAGAACAGC	217
		R CCACCTTGAGAGGGTTGACA	
	CD61	F TGTATGGGACTCAAGATTGGAGAC	187
		R AGCGATGGCTATTAGGTTCAGC	
	p21	F CAGGGGACAGCAGAGGAAGA	189
		R TTAGGGCTTCCTCTTGGAGAA	
	cyclin D1	F CCGTCCATGCGGAAGATC	86
		R ATGGCCAGCGGGAAGAC	
	β-actin	F TGGCATTGCCGACAGGAT	83
		R GCTCAGGAGGAGCAATGATCT	
ChIP	TRPA1 promoter	F TCCGCGTTTCTACCTCCTCTC	134
		R GCGCTGCAGCTCACAGG	

Figure S1

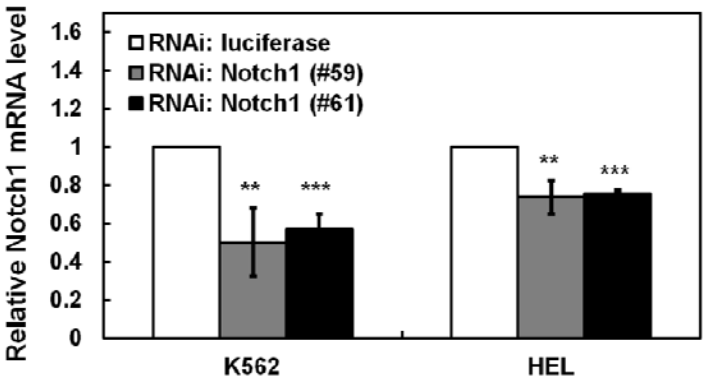

Figure S1. The knockdown of Notch1 receptor in K562 and HEL cells. K562 and HEL cells were transfected with siRNA vectors against Notch1 receptor (#59 and #61) or luciferase for 2 days. The relative transcription levels of Notch1 receptor in the transfected cells were determined by quantitative real-time PCR and normalized to those of β-actin. The means of at least three independent experiments performed in triplicate are shown. **, P <0.01; ***, P <0.001.

Figure S2

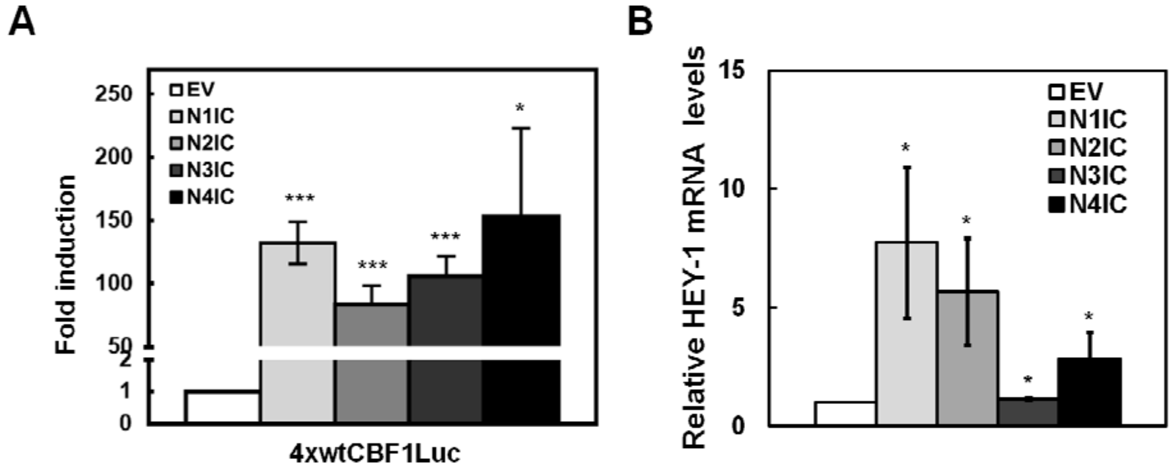

Figure S2. The overexpression of Notch receptor intracellular domains enhance activity of CBF1-dependent promoter and mRNA levels of Notch target HEY-1 in K562 cells. (A) Reporter plasmid 4×wtCBF1Luc containing four copies of wild-type CBF1-response elements was co-transfected with expression constructs of Notch1 receptor (N1IC), Notch2 receptor (N2IC), Notch3 receptor (N3IC), and Notch4 receptor (N4IC) intracellular domains or empty vector (EV) into K562 cells for 48 hours for reporter gene assay. (B) K562 cells were transfected with expression constructs of N1IC, N2IC, N3IC, and N4IC for 2 days. The relative transcription levels of HEY-1 in the transfected cells were determined by quantitative real-time PCR and normalized to those of β-actin. The means of at least three independent experiments performed in triplicate are shown. *, P <0.05; ***, P <0.001.

Figure S3

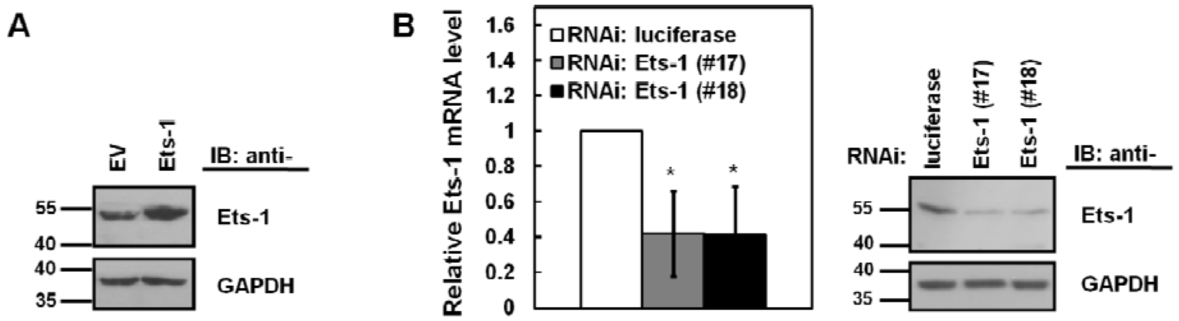

Figure S3. The overexpression and knockdown of Ets-1 in K562 cells. (A) K562 cells were transfected with Ets-1-expressing construct or empty vector (EV) for 2 days. Whole-cell extracts of the transfected cells were prepared for Western blot analysis using anti-Ets-1 and anti-GAPDH antibodies. (B) K562 cells were transfected with siRNA vectors against Ets-1 (#17 and #18) or luciferase for 2 days. The relative transcription levels of Ets-1 in the transfected cells were determined by quantitative real-time PCR and normalized to those of β-actin (*left*). Whole-cell extracts of the transfected cells were prepared for Western blot analysis using anti-Ets-1 and anti-GAPDH antibodies (*right*). The means of at least three independent experiments performed in triplicate are shown. *, $P < 0.05$.

Figure S4

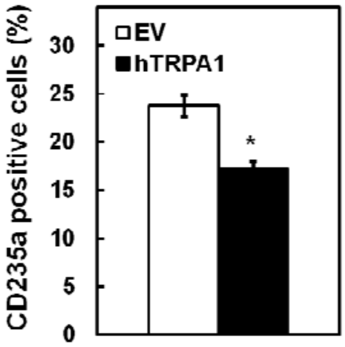

Figure S4. Overexpression of TRPA1 decreased CD235a-positive cells in hemin-treated K562 cells. K562 cells were transfected with TRPA1-expressing construct (hTRPA1) or empty vector (EV) for two days and subsequently treated with 40 μM hemin for 4 days to induce erythroid differentiation. The treated cells were stained with anti-huamn CD235a (glycophorin A)-FITC antibodies and further analyzed CD235a-positive cells by flow cytometry. The means of three independent experiments performed in triplicate are shown. *, P <0.05.

Figure S5

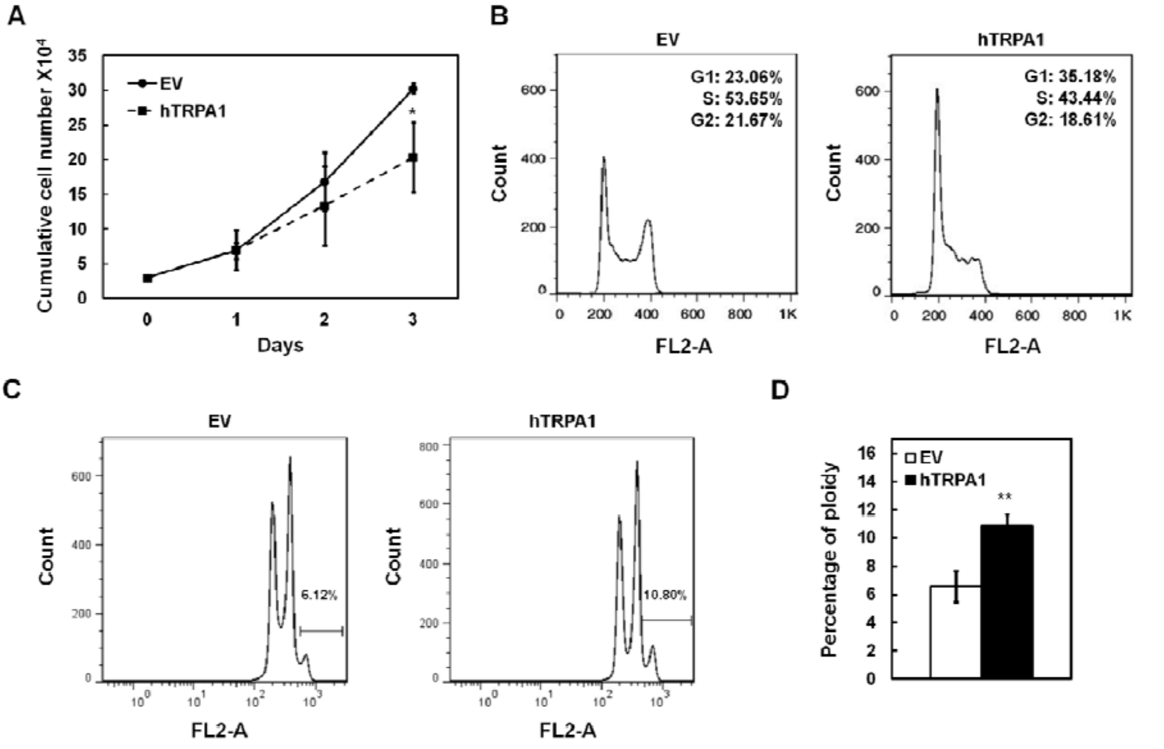

Figure S5. Overexpression of TRPA1 slightly caused the arrest of cell cycle and increase of PMA-mediated polyploidization in K562 cells. K562 cells were transfected with TRPA1-expressing construct (hTRPA1) or empty vector (EV) for two days. (A) The transfected cells were seeded and then counted by trypan blue exclusion method at the time indicated. (B) The transfected cells were stained with propidium iodide to analyze their DNA contents by flow cytometry. (C) Additionally, the transfected cells were also treated with 5 ng/ml PMA for 3 days to induce megakaryocyte differentiation. For a ploidy analysis, the treated cells were stained with propidium iodide to analyze their DNA contents by flow cytometry. The percentage of ploidy was determined by DNA content >4N. The means of

at least three independent experiments performed in triplicate are shown. *, $P < 0.05$; **, $P < 0.01$.

Figure S6

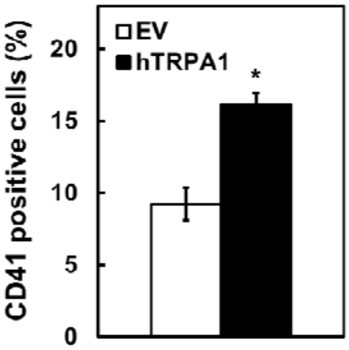

Figure S6. Overexpression of TRPA1 increased CD41-positive cells in PMA-treated K562 cells. K562 cells were transfected with TRPA1-expressing construct (hTRPA1) or empty vector (EV) for two days and subsequently treated with 5 ng/ml PMA for 4 days to induce megakaryocytic differentiation. The treated cells were stained with anti-huamn CD41a-FITC antibodies and further analyzed CD41-positive cells by flow cytometry. The means of three independent experiments performed in triplicate are shown. *, $P < 0.05$.

Figure S7

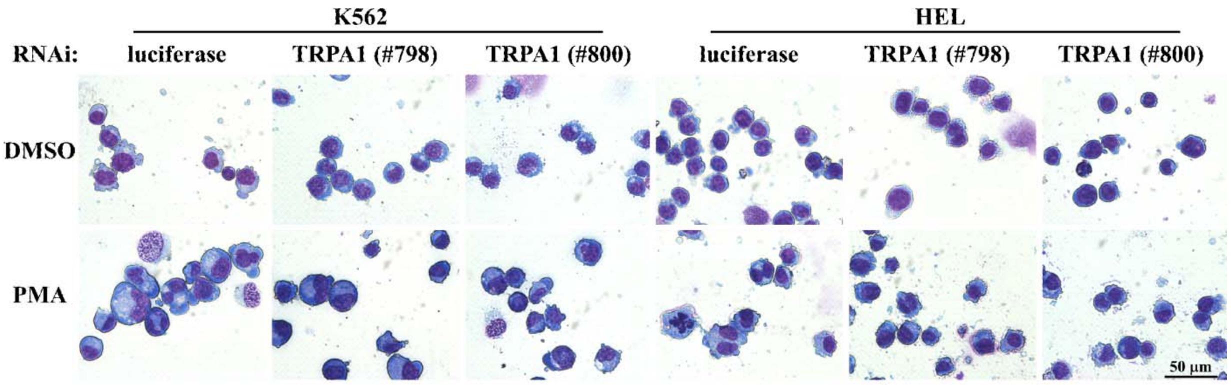

Figure S7. TRPA1 knockdown suppressed PMA-induced-megakaryocyte differentiation of K562 and HEL cells. K562 (*left*) and HEL (*right*) cells were treated with PMA or DMSO for induction of megakaryocyte differentiation after transfection with siRNA vectors against TRPA1 (#798 and #800) or luciferase. Then morphology of the treated cells was examined using light microscopy after Giemsa staining as described in the legends to Fig. 6. Scale bar: 50 μm. Data are representative of 3 experiments.

Figure S8

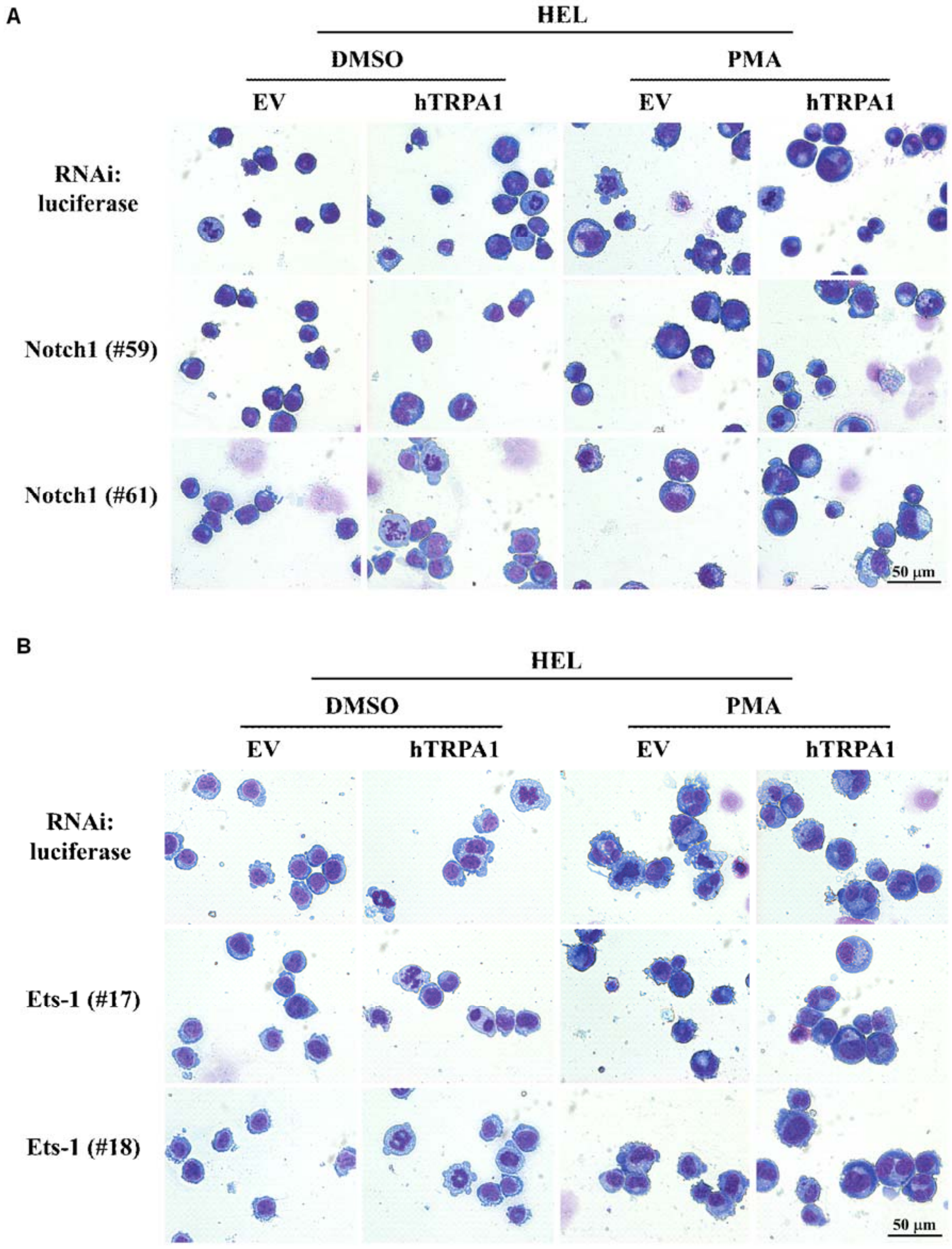

Figure S8. Reduction of megakaryocyte differentiation in HEL cells by Notch1 or Ets-1 knockdown was relieved by TRPA1 overexpression. (A, B) After co-transfection

with TRPA1-expressing construct (hTRPA1) or empty vector (EV) and siRNA vectors against Notch1 receptor (#59 and #61) (A), Ets-1 (#17 and #18) (B), or luciferase, HEL cells were treated with PMA or DMSO for induction of megakaryocyte differentiation and subsequently morphological examination by light microscopy after Giemsa staining as described above. Scale bar: 50 μm. Data are representative of 3 experiments.

Figure S9

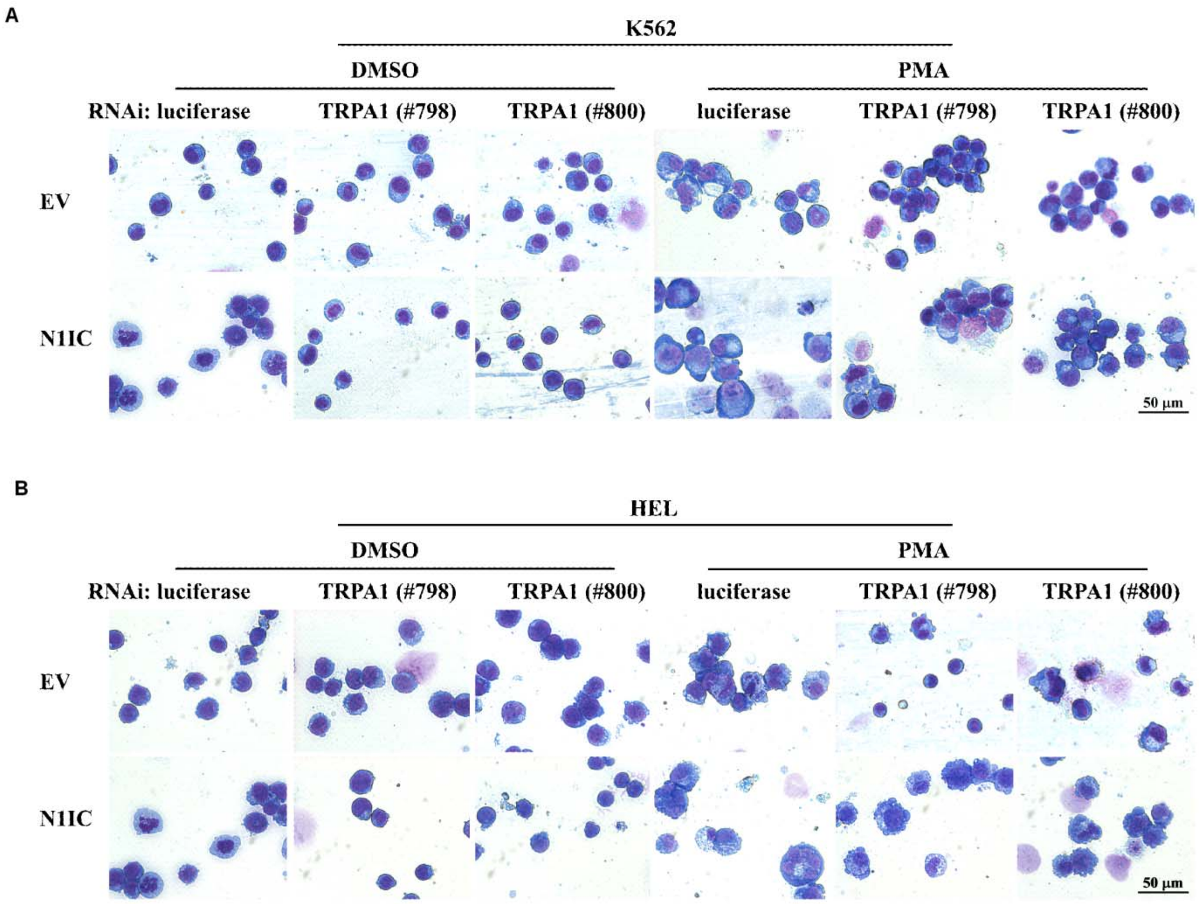

Figure S9. N1IC promoted megakaryocyte differentiation abilities of K562 and HEL cells through TRPA1. (A, B) K562 (A) and HEL (B) cells were co-transfected with expression constructs of N1IC or empty vector (EV) and siRNA vectors against TRPA1 (#798 and #800) or luciferase for 2 days. Then the transfected cells were treated with PMA or DMSO for 2 days to induce megakaryocytic differentiation as described above. The morphological examination of the treated cells was performed by light microscopy after Giemsa staining. Scale bar: 50 μm. Data are from a representative experiment that was performed three times with identical results.

Figure S10

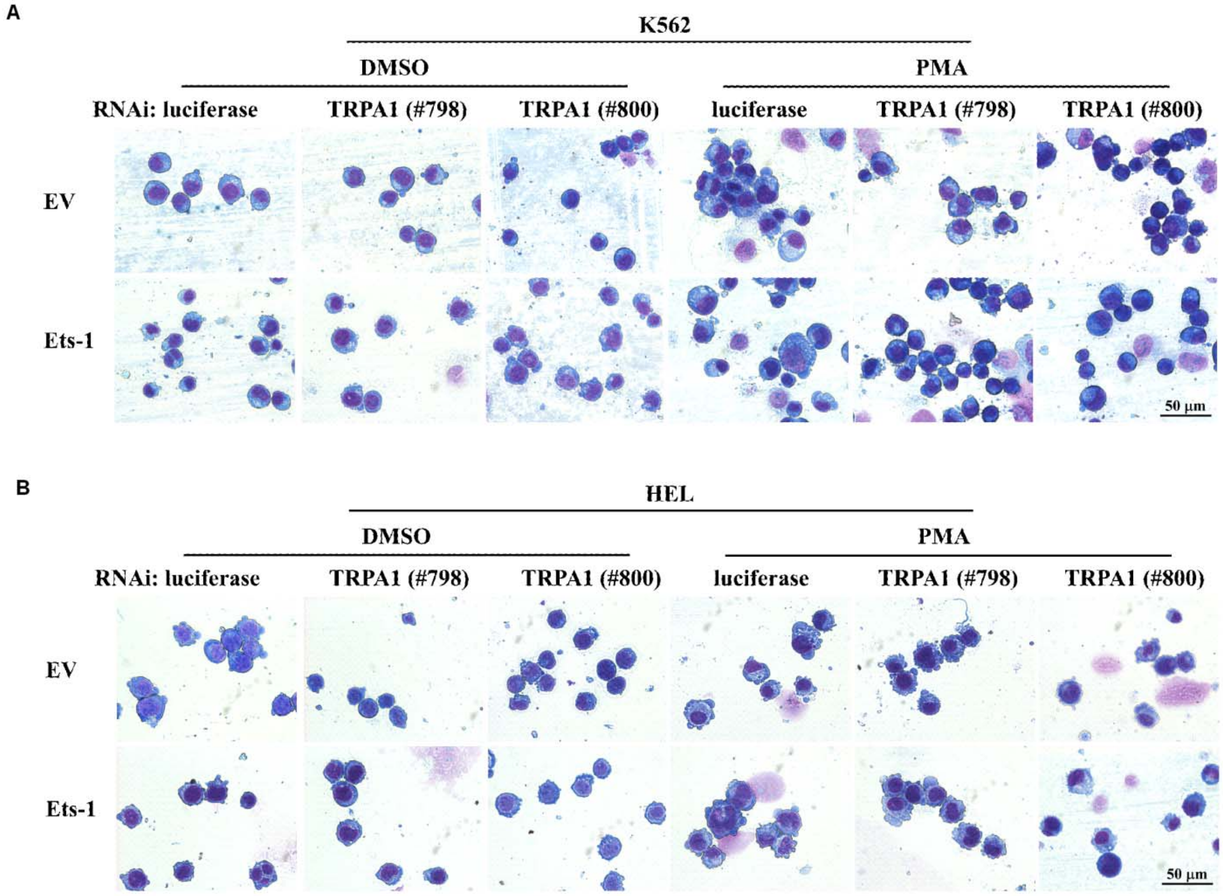

Figure S10. Ets-1 enhanced megakaryocyte differentiation abilities of K562 and HEL cells through TRPA1. (A, B) K562 (A) and HEL (B) cells were co-transfected with expression constructs of Ets-1 or empty vector (EV) and siRNA vectors against TRPA1 (#798 and #800) or luciferase for 2 days. Then the transfected cells were treated with PMA or DMSO for 2 days and then examined megakaryocytic differentiation as described above. Scale bar: 50 μm. Data are representative of 3 experiments.

Figure S11

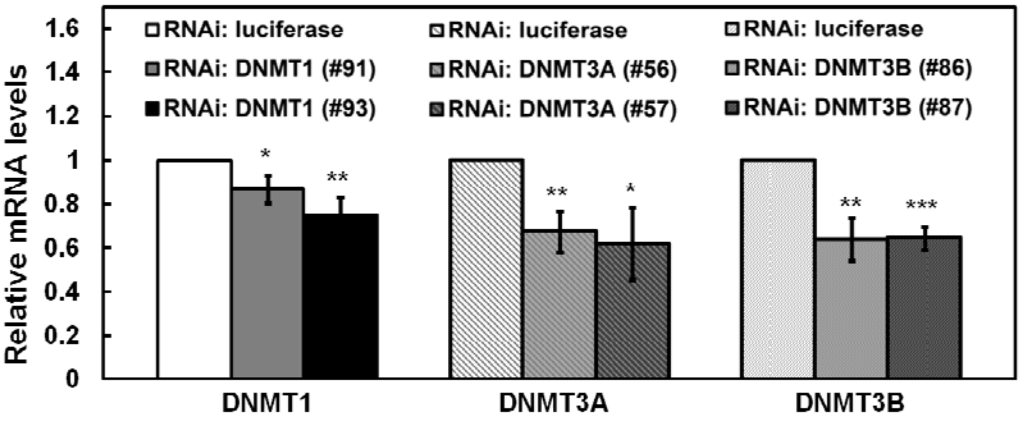

Figure S11. The knockdown of DNMTs in K562 cells. K562 cells were transfected with siRNA vectors against DNMT1 (#91 and #93), DNMT3A (#56 and #57), DNMT3B (#86 and #87) or luciferase for two days. The relative transcription levels of DNMT1, DNMT3A, and DNMT3B in the transfected cells were determined by quantitative real-time PCR and normalized to those of β-actin. The means of at least three independent experiments performed in triplicate are shown. *, P <0.05; **, P <0.01; ***, P <0.001.

Figure S12

Full-length blots of Fig. 1A

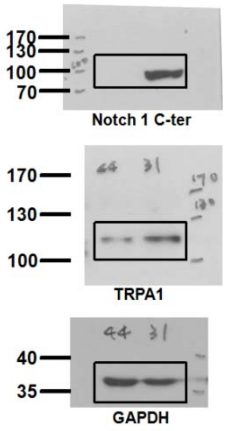

Full-length blots of Fig. 1B

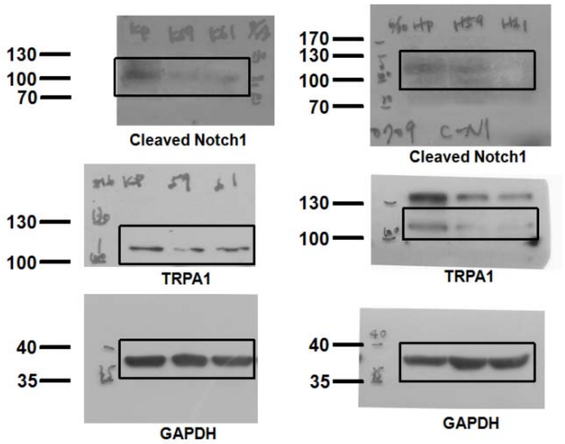

Full-length blots of Fig. 1C

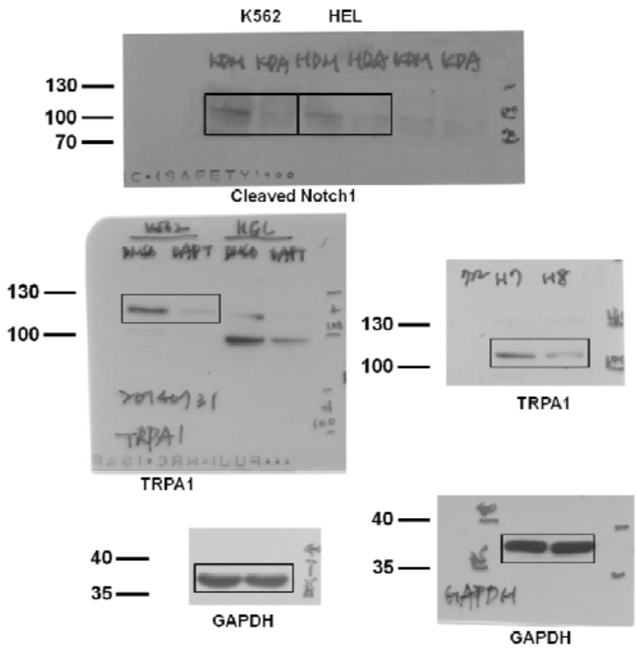

Full-length blots of Fig. 2C

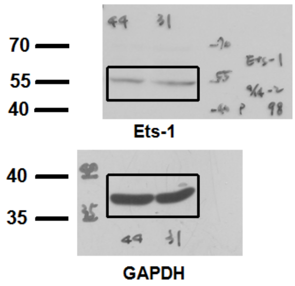

Full-length blots of Fig. 2F

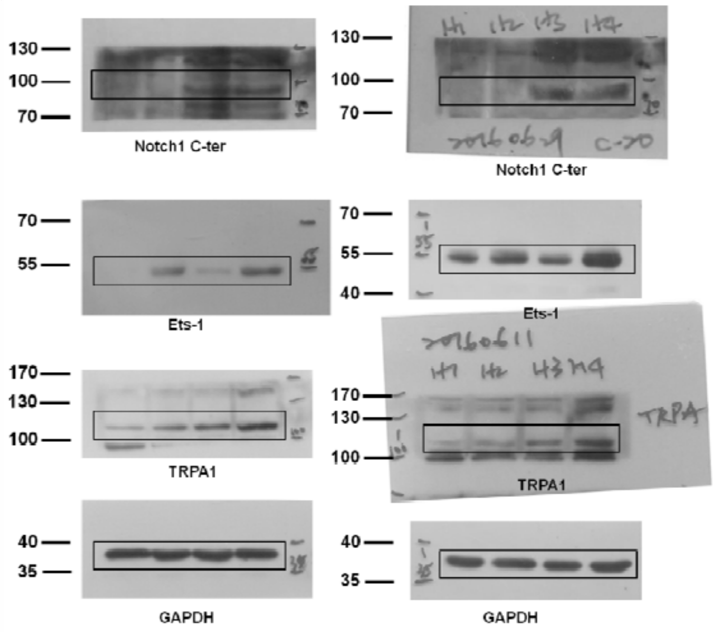

Full-length gel of Fig. 2G

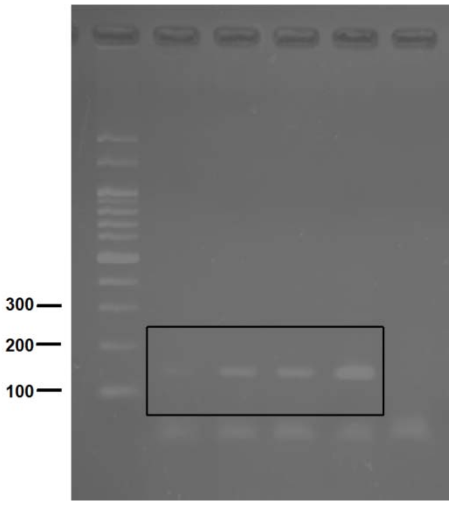

Full-length blots of Fig. 3E

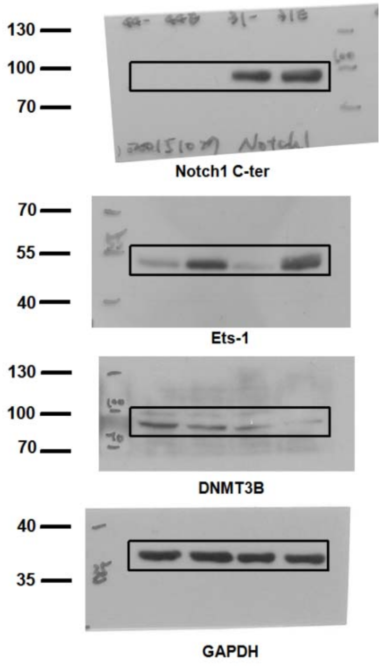

Full-length blots of Fig. 4A

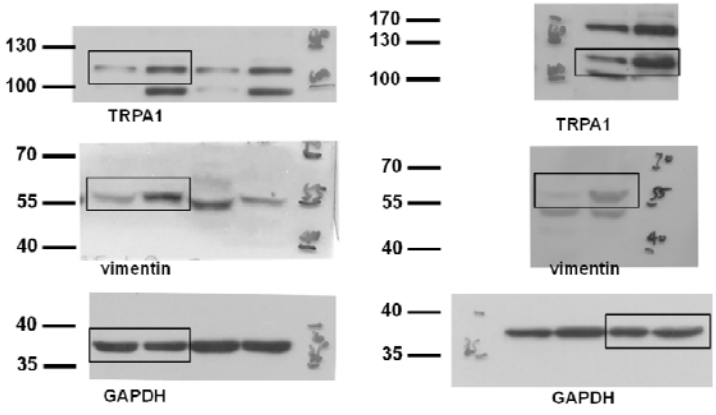

Full-length blots of Fig. 4B

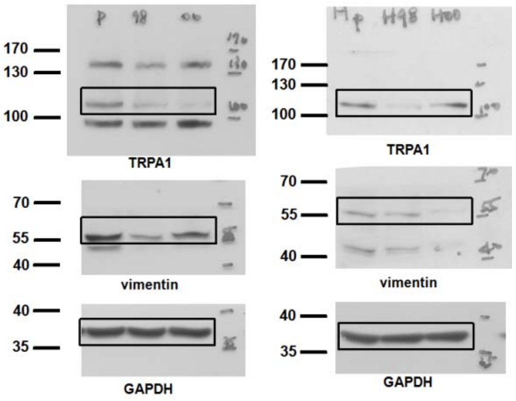

Full-length blots of Fig. 4E

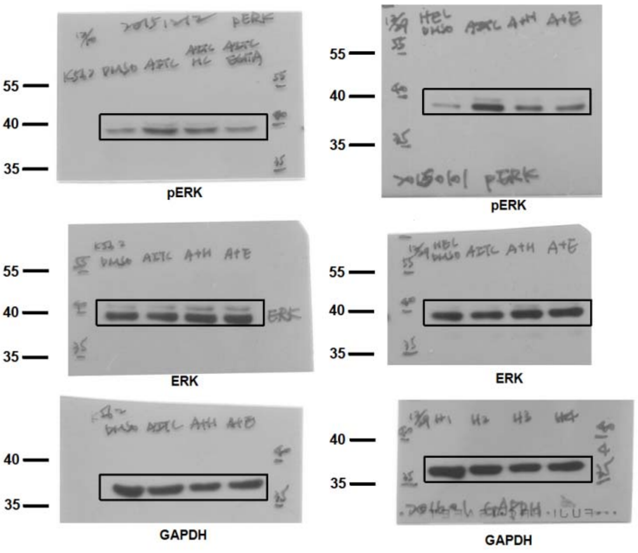

Figure S12. The full-length images shown in main Figure 1 to Figure 4. Some transferred membranes were cut according to molecular weights of the detected proteins before incubation with appropriate antibodies. This is to simultaneously detect several proteins on the same membrane and to economize on the usage of antibodies and reagents.

Figure S13

Original images of Fig. 6C

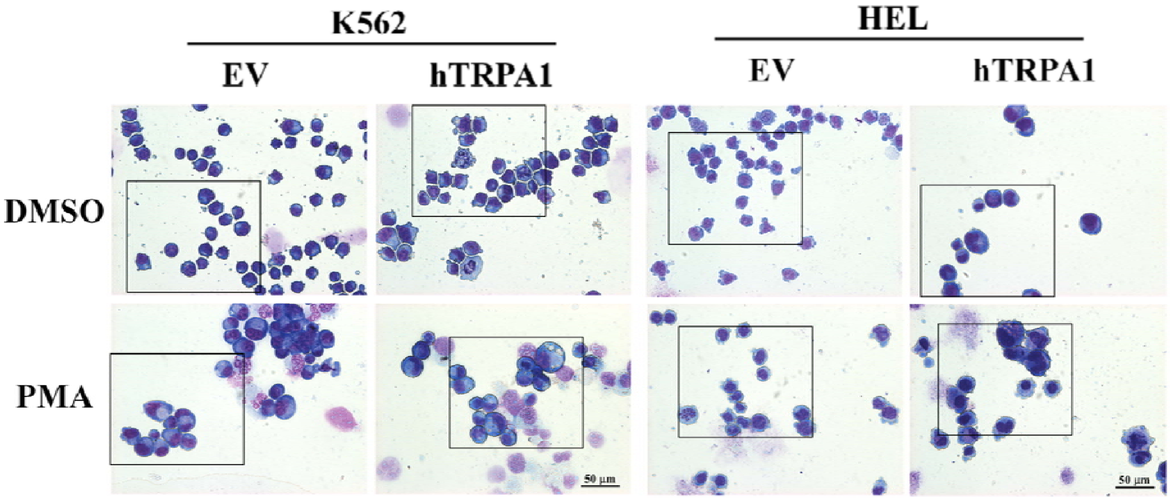

Original images of Fig. 7A

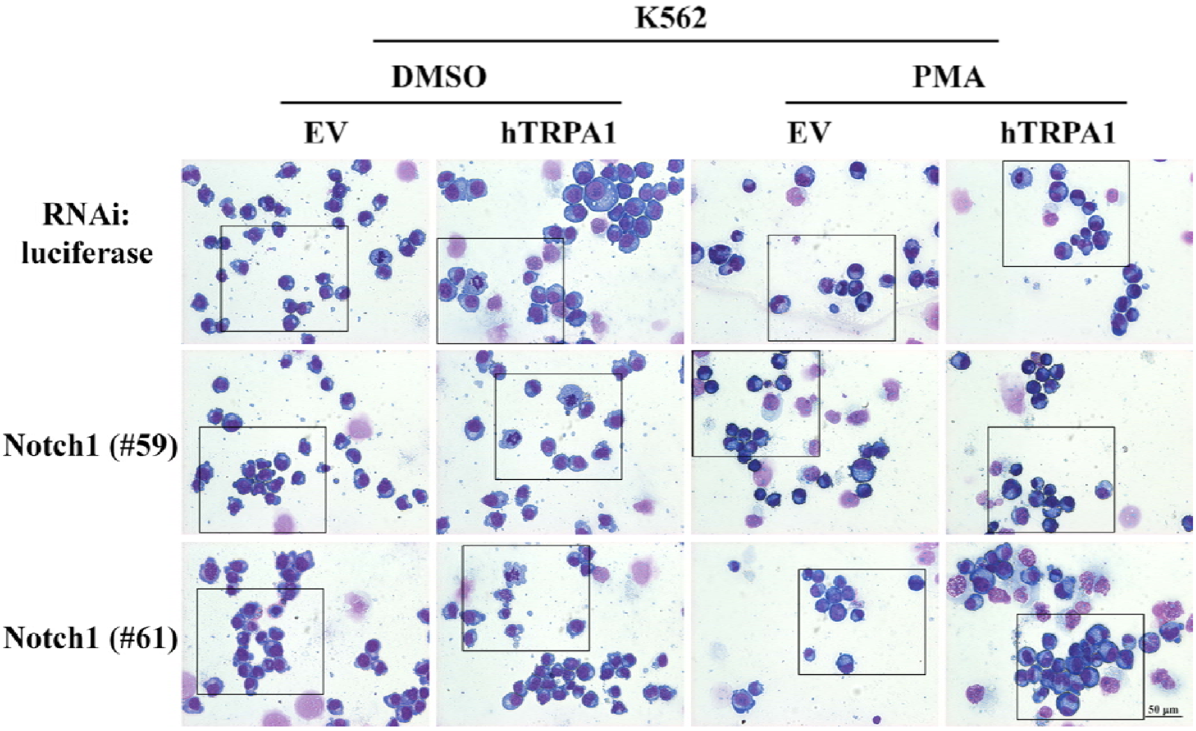

Original images of Fig. 7B

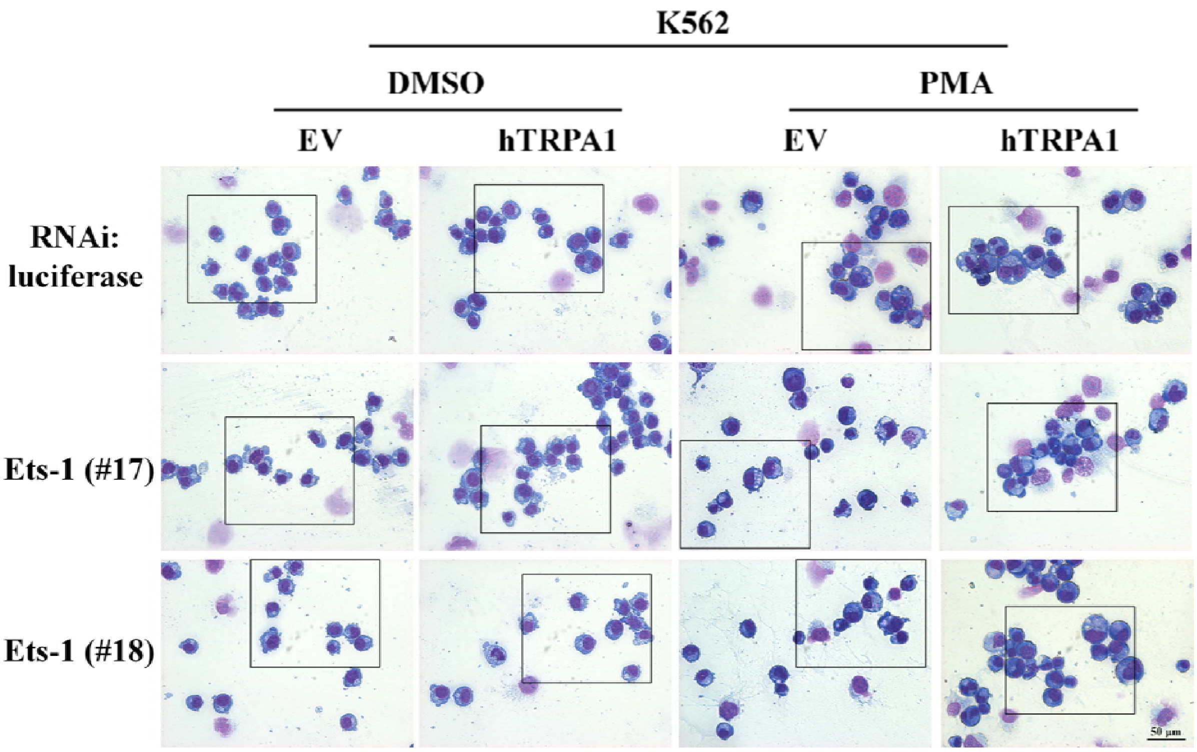

Original images of Fig. 8C

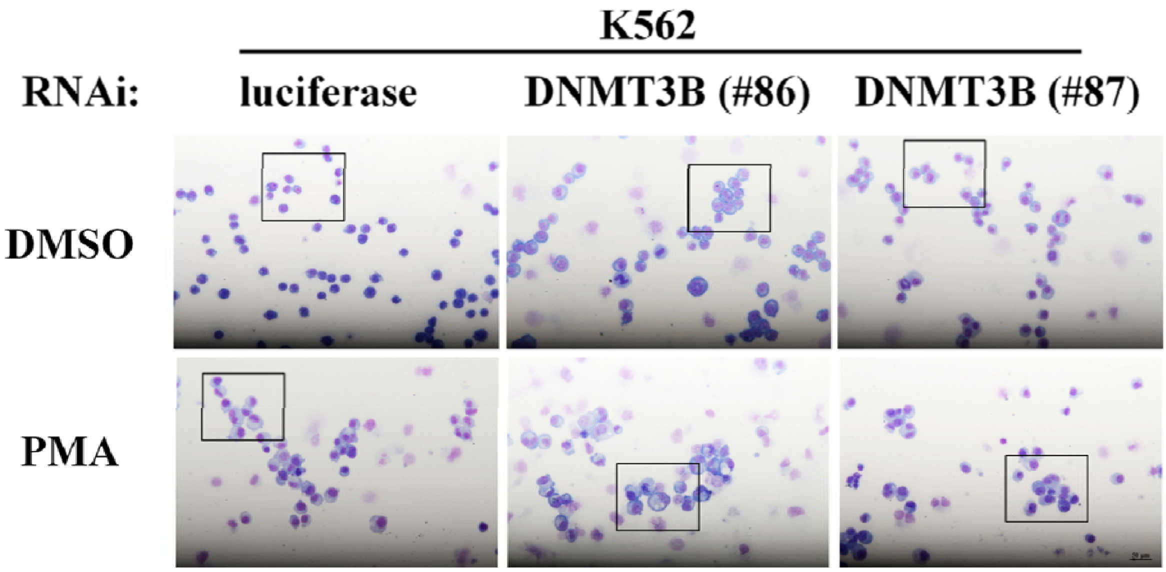

Figure S13. **The original images shown in main Figure 6 to Figure 8.**
